# Constitutional *BRCA1* Methylation and Risk of Incident Triple-Negative Breast Cancer and High-grade Serous Ovarian Cancer

**DOI:** 10.1001/jamaoncol.2022.3846

**Published:** 2022-09-08

**Authors:** Per E. Lønning, Oleksii Nikolaienko, Kathy Pan, Allison W. Kurian, Hans P. Eikesdal, Mary Pettinger, Garnet L. Anderson, Ross L. Prentice, Rowan T. Chlebowski, Stian Knappskog

**Affiliations:** 1 K.G. Jebsen Centre for Genome-Directed Cancer Therapy, Department of Clinical Science, University of Bergen, Bergen, Norway; 2 Department of Oncology, Haukeland University Hospital, Bergen, Norway; 3 Lundquist Institute for Biomedical Innovation at Harbor-UCLA Medical Center, Torrance, California; 4 Departments of Medicine and of Epidemiology and Population Health, Stanford University, Stanford, California; 5 Division of Public Health Sciences Division, Fred Hutchinson Cancer Center, Seattle, Washington

## Abstract

**Importance:**

About 25% of all triple-negative breast cancers (TNBCs) and 10% to 20% of high-grade serous ovarian cancers (HGSOCs) harbor *BRCA1* promoter methylation. While constitutional *BRCA1* promoter methylation has been observed in normal tissues of some individuals, the potential role of normal tissue methylation as a risk factor for incident TNBC or HGSOC is unknown.

**Objective:**

To assess the potential association between white blood cell *BRCA1* promoter methylation and subsequent risk of incident TNBC and HGSOC.

**Design, Setting, and Participants:**

This case-control study included women who were participating in the Women’s Health Initiative study who had not received a diagnosis of either breast or ovarian cancer before study entrance. A total of 637 women developing incident TNBC and 511 women developing incident HGSOC were matched with cancer-free controls (1841 and 2982, respectively) in a nested case-control design. Cancers were confirmed after central medical record review. Blood samples, which were collected at entry, were analyzed for *BRCA1* promoter methylation by massive parallel sequencing. The study was performed in the Mohn Cancer Research Laboratory (Bergen, Norway) between 2019 and 2022.

**Main Outcomes and Measures:**

Associations between *BRCA1* methylation and incident TNBC and incident HGSOC were analyzed by Cox proportional hazards regression.

**Results:**

Of 2478 cases and controls in the TNBC group and 3493 cases and controls in the HGSOC group, respectively, 7 (0.3%) and 3 (0.1%) were American Indian or Alaska Native, 46 (1.9%) and 30 (0.9%) were Asian, 1 (0.04%) and 1 (0.03%) was Native Hawaiian or Pacific Islander, 326 (13.2%) and 125 (3.6%) were Black or African, 56 (2.3%) and 116 (3.3%) were Hispanic, 2046 (82.6%) and 3257 (93.2%) were White, and 35 (1.4%) and 35 (1.0%) were multiracial. Median (range) age at entry was 62 (50-79) years, with a median interval to diagnosis of 9 (TNBC) and 10 (HGSOC) years. Methylated *BRCA1* alleles were present in 194 controls (5.5%). Methylation was associated with risk of incident TNBC (12.4% methylated; HR, 2.35; 95% CI, 1.70-3.23; *P* < .001) and incident HGSOC (9.4% methylated; HR, 1.93; 95% CI, 1.36-2.73; *P* < .001). Restricting analyses to individuals with more than 5 years between sampling and cancer diagnosis yielded similar results (TNBC: HR, 2.52; 95% CI, 1.75-3.63; *P* < .001; HGSOC: HR, 1.82; 95% CI, 1.22-2.72; *P* = .003). Across individuals, methylation was not haplotype-specific, arguing against an underlying cis-acting factor. Within individuals, *BRCA1* methylation was observed on the same allele, indicating clonal expansion from a single methylation event. There was no association found between *BRCA1* methylation and germline pathogenic variant status.

**Conclusions and Relevance:**

The results of this case-control suggest that constitutional normal tissue *BRCA1* promoter methylation is significantly associated with risk of incident TNBC and HGSOC, with potential implications for prediction of these cancers. These findings warrant further research to determine if constitutional methylation of tumor suppressor genes are pancancer risk factors.

## Key Points

**Question:**

Is mosaic *BRCA1* promoter methylation in normal tissue associated with the risk of incident high-grade serous ovarian cancer (HGSOC) and triple-negative breast cancer (TNBC)?

**Findings:**

In this nested case-control study including 637 women developing TNBC and 511 developing HGSOC, white blood cell *BRCA1* promoter methylation was associated with a significantly elevated risk of developing both cancer forms (hazard ratio for HGSOC of 1.93 and TNBC of 2.35). The results remained significant in a subgroup analysis of women who received a diagnosis of cancer more than 5 years after blood sampling (hazard ratio of 1.82 and 2.52, respectively).

**Meaning:**

The study results may serve as proof of concept for early life constitutional methylation as a cancer risk factor.

## Teaser

This case-control study examines the potential association between white blood cell *BRCA1* promoter methylation and subsequent risk of incident triple-negative breast cancer and high-grade serous ovarian cancer.

## Introduction

Women with breast cancer type 1 susceptibility gene (*BRCA1*) germline pathogenic variants are at high risk of triple-negative breast cancer (TNBC) and high-grade serous ovarian cancer (HGSOC).^1^ *BRCA1*, like *BRCA2* and several other genes, is required for homologous recombination repair of double-stranded DNA breaks.^2^ While most TNBCs and HGSOCs have gene expression profiles indicating homologous recombination deficiency (HRD),^3,4,5^ in many cases, no genetic alteration explains the HRD-positive status.

An alternative mechanism to HRD-positive status is *BRCA1* promoter methylation, which causes downregulation of transcription from the *BRCA1* gene.^6,7^ *BRCA1* methylation has been detected in tumors of 25% to 30% of TNBCs and 10% to 20% of HGSOCs and has been associated with HRD mutational and gene expression signatures, such as those in cancers in *BRCA1* germline pathogenic variant carriers.^3,4,5,8^ While *BRCA1* methylation is commonly considered to have somatic origin,^9^ the HRD mutational signatures indicate that *BRCA1* methylation may occur early in tumor evolution, potentially even as a first triggering event in certain TNBC and HGSOC cases.

Constitutional epimutations are aberrant normal tissue methylation events occurring in utero.^10^ While several studies have assessed white blood cell (WBC) *BRCA1* methylation as a potential risk factor for TNBC or HGSOC,^11,12,13,14,15,16,17^ apart from 1 study in TNBC^18^ and 1 in HGSOC,^19^ these studies have provided mixed results because of a limited number of cases and lack of statistical power. However, the main limitation, which was shared by all prior studies, was blood sample collection after cancer diagnosis with potential for disease-related confounding. Consequently, to our knowledge, associations between constitutional *BRCA1* methylation and incident TNBC and HGSOC have not been definitively assessed.

In this article, we assessed the potential association of antecedent WBC *BRCA1* promoter methylation with incident TNBC and HGSOC among Women’s Health Initiative (WHI) study participants in a nested case-control study. These tumor forms were selected based on their association with *BRCA1* germline pathogenic variants and previous findings of elevated *BRCA1* methylation among individuals with a diagnosis of HGSOC but not among other ovarian cancers.^19^

## Methods

### Study Design

The WHI study design details were reported previously.^20^ Briefly, 161 808 postmenopausal women aged 50 to 79 years with an anticipated survival of longer than 3 years were recruited from 40 US clinical centers between 1993 and 1998 to participate in either an observational study or 1 or more of 4 clinical trials (Supplement 1). At study entry, self-administered questionnaires were used to collect demographic characteristics and medical, reproductive, and family history. Race and ethnicity were determined by participant self-report against fixed categories. Entry blood samples were obtained after at least 12 hours of fasting via a prespecified protocol standardized across all study sites. Samples were shipped on dry ice and stored at −80 °C at Fisher Bioservices (Rockville, Maryland).

Clinical outcomes were ascertained annually from enrollment in the observational study and every 6 months for clinical trial participants during the 8.5-year intervention period and annually thereafter. Self-reported cancers were initially confirmed with medical record review at the clinical centers by trained physician adjudicators, with final confirmation at the clinical coordinating center.

Participants provided written informed consent and study protocols were approved at all clinical centers. The current study was additionally approved by the regional ethical committee of the Western Norwegian Health Region.

For the current study, all WHI participants with incident TNBC or HGSOC were included (Supplement 1). Women reporting a history of breast or ovarian cancer at baseline were excluded. For the TNBC study, women with a previous unilateral or bilateral mastectomy for any reason were excluded, while women reporting previous oophorectomy were excluded from the HGSOC study.

**Figure 1.**
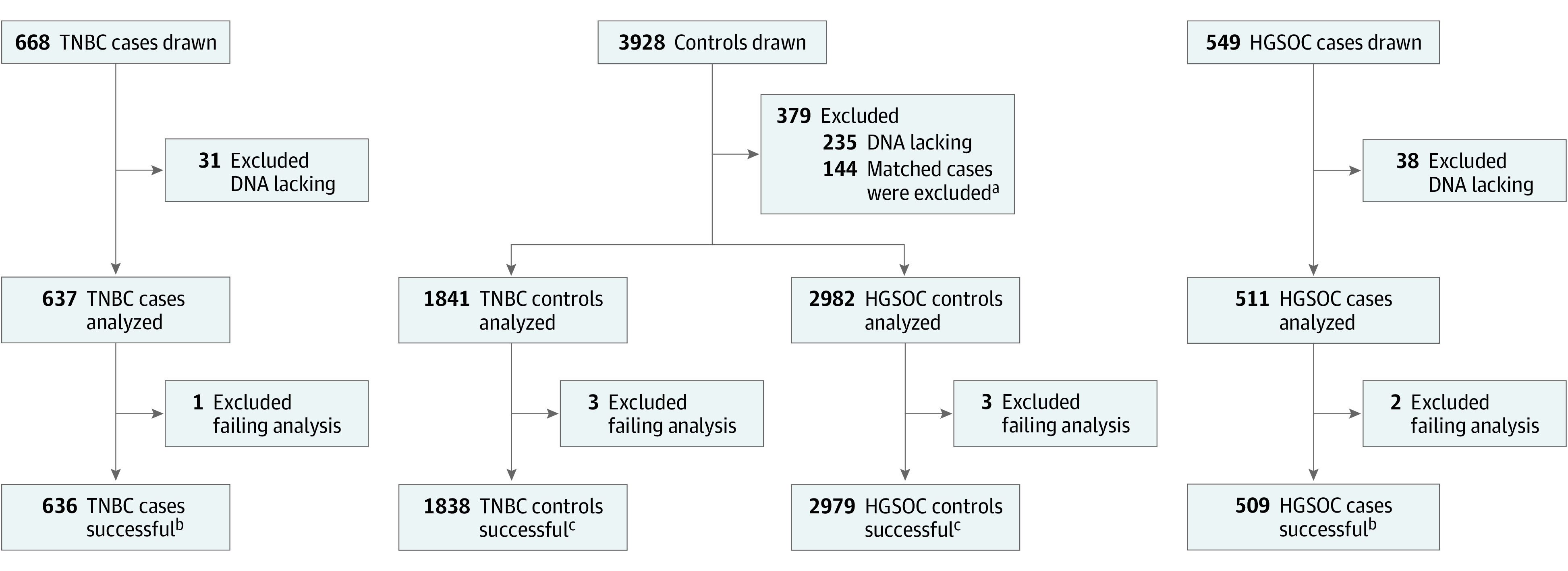
Flowchart Depicting Samples Drawn and Successfully Analyzed From Patients and Controls ^a^A total of 144 controls were excluded because their matched cases had too low DNA concentration. ^b^Five cases had triple-negative breast cancer (TNBC) and high-grade serous ovarian cancer (HGSOC) and were included as cases in the hazard ratio estimates for TNBC and HGSOC. ^c^A total of 1272 controls were included as controls in the hazard ratio estimates for TNBC and HGSOC.

The study was conducted as 2 nested case-control studies. Based on up-front statistical power calculations (eMethods in Supplement 2), women with incident TNBC and controls were matched at a 1:3 ratio, whereas women with incident HGSOC and controls were matched at a 1:6 ratio. Controls were matched for age at entry, hormone therapy use, race and ethnicity, and DNA extraction method. In addition, TNBC cases and controls were matched based on age at prior bilateral oophorectomy. Further, controls were required to be alive and disease free at the time of the case diagnosis (eMethods in Supplement 2). The statistical analyses included samples with *BRCA1* promoter methylation determinations from incident TNBC (n = 637), incident HGSOC (n = 511), and matched cancer-free controls (n = 1841 and 2982, respectively), as depicted in Figure 1. To limit the required sample number, 1274 of the controls (35.9%) were included in the TNBC and HGSOC groups. Controls were first drawn for the HGSOC comparison and were then eligible to be selected as controls for the TNBC cases. Five cases had TNBC and HGSOC and were included in the risk assessments for both cancers. The study was conducted according to Strengthening the Reporting of Observational Studies in Epidemiology (STROBE) reporting guidelines.

### *BRCA1* Promoter Methylation Analysis

A detailed description of the methylation analysis is given in eFigure 1 and eTables 1 and 2 in Supplement 2. In brief, WBC genomic DNA was bisulfite converted, and 4 overlapping regions covering the *BRCA1* promoter area were amplified, pooled, indexed, and sequenced to a very high depth (>20 000×) using the Illumina MiSeq System. Methylation status was scored by predefined criteria as variant epiallele frequency (VEF), referring to the frequency of hypermethylated epialleles (eFigures 2 and 3 in Supplement 2).^21^

All samples were analyzed masked to case-control status. The main cutoff value for methylation positivity was computationally defined based on the assay sensitivity and VEF probability density across the entire sample set, which was masked to case-control status (eFigure 4 in Supplement 2).

### Determining Allele Specificity of Methylation

The region covered by sequencing contains a highly prevalent single-nucleotide polymorphism (SNP), rs799905, which is located in the *BRCA1* gene body (eFigure 1 in Supplement 2). By analyzing methylation in sequencing reads covering this SNP, we assessed the potential allele specificity of methylation in individuals heterozygous for the SNP.

### Association Between *BRCA1* Methylation Status and Cancer Risk Genes

We tested for the association of *BRCA1* methylation status with germline pathogenic variants in a subgroup of 234 participants (5.0%) from the study (173 cases and 61 controls) who had previously been tested for germline pathogenic variants in *BRCA1/2* and in 26 additional cancer risk genes (eTable 7 in Supplement 2) as part of another WHI ancillary study.^22^

### Statistical Methods

The potential associations between *BRCA1* methylation and incident TNBC and HGSOC were assessed by estimating hazard ratios (HRs) and 95% CIs. The HRs were determined using Cox proportional hazards regression in matched case-control groups, including age, race and ethnicity, previous hormone use, DNA extraction method, and (for TNBC) previous oophorectomy as covariates. In addition, we performed hypothesis-generating supportive subgroup analyses.

Power estimates based on previous results of methylation frequency among HGSOC cases and controls^19^ are outlined in the eMethods in Supplement 2. In brief, a conservative assumption of 600 TNBC cases and 400 HGSOC cases was made. Matching 600 TNBC with 1800 control samples in a nested design provides a power of 0.88 to detect an HR of 2.0. Similarly, for HGSOC, comparing 400 patients with 2400 controls provides a power of 0.80.

In sensitivity analyses, we analyzed HR associated with *BRCA1* methylation status in different promoter subregions, age groups, cutoff levels for methylation positivity, and methylation positivity assessed by methylation beta values (ratio of methylated to total number of cytosines; eMethods in Supplement 2). As oophorectomy has been associated with a reduced risk of TNBC in *BRCA1* germline pathogenic variant carriers,^23^ we also performed subgroup analysis assessing HRs for TNBC among cases and controls who were not undergoing an oophorectomy. Analysis were conducted using R, version 4.0.3 (R Foundation).

## Results

The demographic characteristics and flowchart of TNBC and HGSOC cases and controls are presented in the Table and Figure 1. There was no systemic difference between individuals excluded from analysis because of lack of DNA and final participants. *BRCA1* methylation status was not associated with a self-reported family history of either breast or ovarian cancer (eTable 3 in Supplement 2). Among controls, across all age groups, 194 (5.5%) had methylated *BRCA1* alleles (eTable 4 in Supplement 2), contrasting with 79 (12.4%) and 48 (9.4%) in TNBC and HGSOC cases, respectively. For cases and controls, most alleles were either fully methylated or unmethylated (eFigures 2 and 3 in Supplement 2).

**Table.** Demographic Characteristics of Patients and Controls Included in the Trial

Characteristic	No. (%)			
	TNBC		HGSOC	
	Cases (n = 637)	Controls (n = 1841)^a^	Cases (n = 511)	Controls (n = 2982)^a^
Age, y				
Mean (SD)	62.1 (6.81)	62.1 (6.74)	62.3 (6.42)	62.7 (6.82)
Median (range)	62.0 (50.0-78.0)	62.0 (50.0-79.0)	62.0 (50.0-79.0)	62.0 (50.0-79.0)
IQR (Q1-Q3)	11.0 (57.0-68.0)	10.0 (57.0-67.0)	9.50 (57.5-67.0)	11.0 (57.0-68.0)
Ethnicity				
Not Hispanic or Latino	621 (97.5)	1795 (97.5)	493 (96.5)	2880 (96.6)
Hispanic or Latino	13 (2.0)	43 (2.3)	16 (3.1)	100 (3.4)
Unknown or not reported	3 (0.5)	3 (0.2)	2 (0.4)	2 (0.1)
Race				
American Indian or Alaska Native	1 (0.2)	6 (0.3)	2 (0.4)	1 (0.0)
Asian	13 (2.0)	33 (1.8)	5 (1.0)	25 (0.8)
Native Hawaiian or other Pacific Islander	0	1 (0.1)	0	1 (0.0)
Black or African American	81 (12.7)	245 (13.3)	18 (3.5)	107 (3.6)
White	525 (82.4)	1521 (82.6)	479 (93.7)	2778 (93.2)
Multiracial	12 (1.9)	23 (1.2)	3 (0.6)	32 (1.1)
Unknown or not reported	5 (0.8)	12 (0.7)	4 (0.8)	38 (1.3)
Years from DNA sampling to diagnosis				
Mean (SD)	10.1 (5.23)	NA	10.4 (5.80)	NA
Median (range)	9.00 (0-23.0)	NA	10.0 (0-23.0)	NA
IQR (Q1-Q3)	8.00 (6.00-14.0)	NA	9.00 (6.00-15.0)	NA
Missing	0	1841 (100)	0	2982 (100)
Bilateral oophorectomy				
No	509 (79.9)	1493 (81.1)	511 (100)	2982 (100)
Yes	118 (18.5)	320 (17.4)	0	0
Missing	10 (1.6)	28 (1.5)	0	0
Family history of breast cancer				
No	158 (24.8)	519 (28.2)	137 (26.8)	857(28.7)
Yes	154 (24.2)	294 (16.0)	94 (18.4)	514 (17.2)
Missing	325 (51.0)	1028 (55.8)	280 (54.8)	1611 (54.0)
Family history of ovarian cancer				
No	289 (45.4)	723 (39.3)	209 (40.9)	1252 (42.0)
Yes	18 (2.8)	42 (2.3)	13 (2.5)	61 (2.0)
Do not know	16 (2.5)	64 (3.5)	17 (3.3)	86 (2.9)
Missing	314 (49.3)	1012 (55.0)	272 (53.2)	1583 (53.1)
High-risk germline variants				
* BRCA1*	2 (0.3)	0	1 (0.2)	0
* BRCA2*	1 (0.2)	0	1 (0.2)	0
No variant detected	101 (15.9)	25 (1.4)	4 (0.8)	36 (1.2)
Missing	533 (83.7)	1816 (98.6)	505 (98.8)	2946 (98.8)

Abbreviations: HGSOC, high-grade serous ovarian cancer; NA, not applicable; Q, quartile; TNBC, triple-negative breast cancer.

^a^ A total of 1274 controls were included as controls for comparison with TNBC and HGSOC. Among these 1274, 2 failed analyses. Thus, 1272 controls were included in the final hazard ratio estimates for TNBC and HGSOC (Figure 1).

### Risk of TNBC and HGSOC

**Figure 2.**
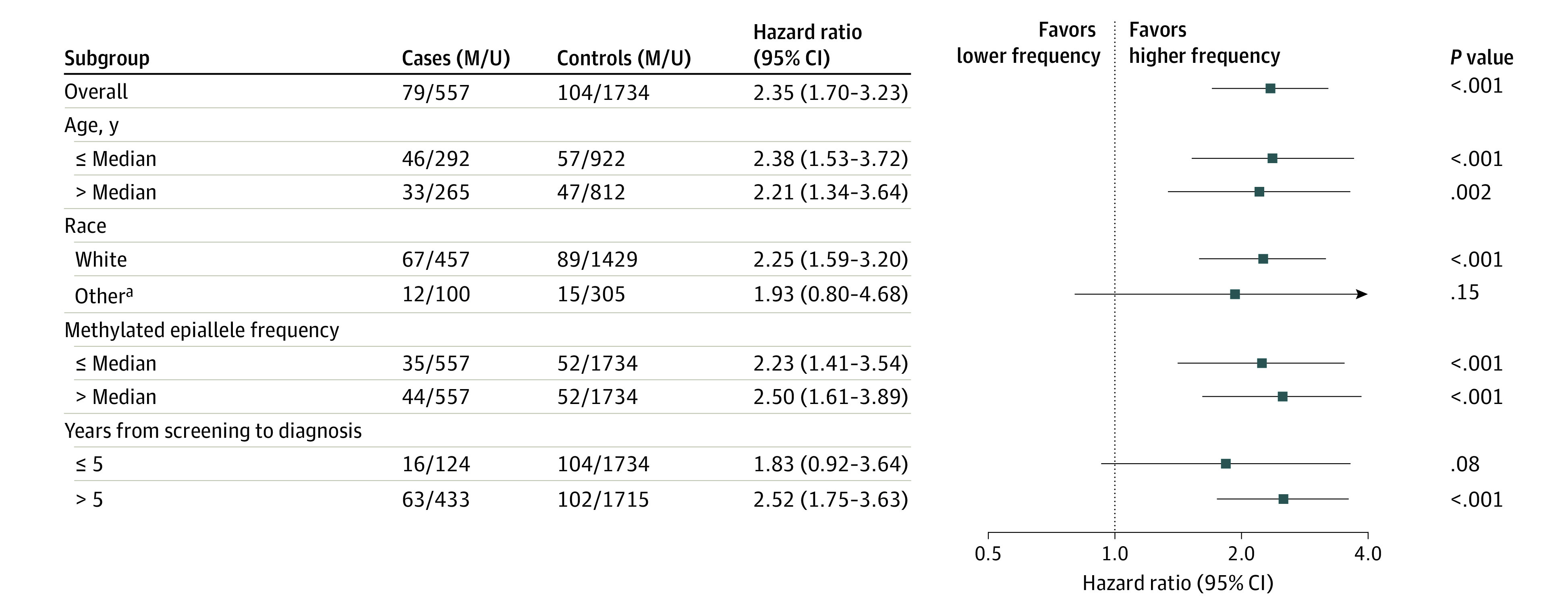
Hazard Ratios for Triple-Negative Breast Cancer (TNBC) Associated With the Presence of Methylated *BRCA1* Alleles in the Overall Cohort and Selected Subgroups M and U represent the number of methylated and unmethylated samples, respectively. Median methylated epiallele frequency equals median variant epiallele frequency for samples classified as methylation-positive. ^a^Other race includes American Indian or Alaska Native, Asian, Native Hawaiian or Pacific Islander, Black or African American, multiracial, unknown, or not reported.

For women with incident TNBC, the median (IQR) follow-up from sampling to diagnosis was 9 (8) years. The presence of methylated *BRCA1* alleles in WBCs was significantly associated with incident TNBC risk (HR, 2.35; 95% CI, 1.70-3.23; *P* < .001; Figure 2). The HR was 1.83 (95% CI, 0.92-3.64; *P* = .08) for TNBC diagnosed 5 years before or less and 2.52 (95% CI, 1.75-3.63; *P* < .001) for those who received a diagnosis more than 5 years after blood sampling. There was no difference between subgroups defined by age at entry or methylation level (Figure 2).

**Figure 3.**
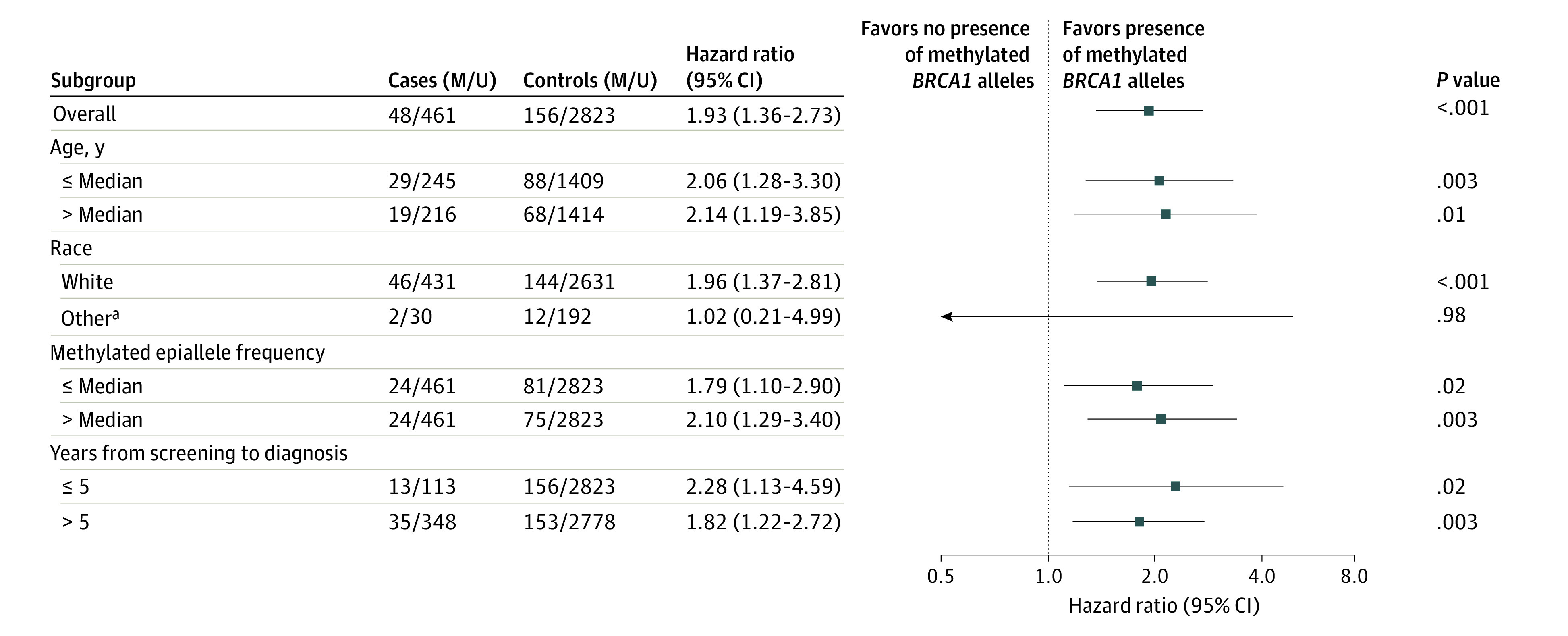
Hazard Ratios for High-grade Serous Ovarian Cancer (HGSOC) Associated With the Presence of Methylated *BRCA1* Alleles in the Overall Cohort and Selected Subgroups M and U represent the number of methylated and unmethylated samples, respectively. Median methylated epiallele frequency here equals median variant epiallele frequency for samples classified as methylation-positive. ^a^Other race includes American Indian or Alaska Native, Asian, Native Hawaiian or Pacific Islander, Black or African American, multiracial, unknown, or not reported.

For women with incident HGSOC, the median (IQR) time from sampling to diagnosis was 10 (9) years. Like the findings for TNBC, the presence of methylated *BRCA1* alleles in WBCs was significantly associated with incident HGSOC risk (HR, 1.93; 95% CI, 1.36-2.73; *P* < .001; Figure 3). The HR was significant for HGSOC diagnosed 5 years or less (HR, 2.28; 95% CI, 1.13-4.59; *P* = .02) or more than 5 years (HR, 1.82; 95% CI, 1.22-2.72; *P* = .003) after blood sampling. Like for TNBC, the association remained significant in subgroups stratified for age at inclusion and methylation level (Figure 3).

### Sensitivity Analysis

We assessed the potential effect of promoter area selection, analytical cutoff, and different methods for methylation classification. These analyses revealed results that confirmed the findings from the main analysis (eFigures 5-14 in Supplement 2). Subgroup analysis excluding TNBC cases and controls who underwent oophorectomy or the 5 cases who had TNBC and HGSOC after DNA collection had no significant association with HRs (eFigure 15 in Supplement 2).

### Allele-Specific *BRCA1* Methylation

Assessing methylation in the region harboring *BRCA1* SNP rs799905, we found methylation frequencies to be similar among individuals carrying the different rs799905 alleles. This suggested that *BRCA1* methylation is not associated with a cis-acting factor (factor located on the same allele as the methylation). However, in individuals who were heterozygous for rs799905 for whom allele-spesific methylation could be determined, the intraindividual methylation was strongly enriched at a single allele. In more than 90% of individuals, more than 95% of the methylation was associated with one of the rs799905 alleles, indicating that *BRCA1* methylation may have occurred as a single, early event that was followed by clonal expansion of the methylated cell (eFigure 16 in Supplement 2).

### Association of *BRCA1* Methylation With Germline Pathogenic Variant Status

*BRCA1* methylation was not associated with germline mutation status for either *BRCA1* or *BRCA2*. Similarly, methylation was not associated with the germline status of any of the 26 other cancer risk genes analyzed (eTable 7 in Supplement 2).

## Discussion

In a nested case-control design in postmenopausal WHI participants, including women with incident TNBC (n = 637), incident HGSOC (n = 511), and matched cancer-free controls (n = 1841 and 2982, respectively), WBC *BRCA1* promoter methylation was significantly associated with higher risk of incident TNBC and incident HGSOC. The association was also significant in analyses restricted to cancers diagnosed more than 5 years after sampling. While an association between *BRCA1* methylation in normal tissue and TNBC and HGSOC was established previously,^11,18,19^ these studies were performed on normal tissue obtained after the patients received their cancer diagnoses. Thus, this study’s findings represent a potential conceptual breakthrough, the results suggesting that *BRCA1* normal tissue methylation in association with TNBC and HGSOC occurs before, and not because of, cancer development. The association of *BRCA1* normal tissue methylation with higher risk for TNBC and HGSOC, the 2 major cancers associated with germline *BRCA1* pathogenic variants, supports this conclusion. Given the frequency of *BRCA1* methylation in TNBC and HGSOC, *BRCA1* normal tissue methylation may be an underlying cause of a substantial fraction of TNBC and HGSOC cases.

Constitutional epimutations may be classified as primary epimutations (methylation in absence of any genetic aberration) or secondary epimutations that are associated with rare germline genetic variants.^10,24^ Secondary epimutations have been detected in only a few cases associated with the Lynch syndrome–associated *MLH1* gene, but also *BRCA1*.^24,25^ In such cases, the fraction of methylated alleles is typically around 50% (ie, 100% of cells carrying the epigenetic variant in a heterozygous individual), and cancer penetrance is high. In contrast, primary *BRCA1* normal tissue methylation assessed in WBCs is not a rare event; it occurs as a low-mosaic phenomenon in 4% to 10% of adult women and newborn girls without cancer.^19,26^ A potential pathogenic role of mosaic methylations may be paralleled with an elevated cancer risk associated with mosaic germline pathogenic variants in *BRCA1* as well as other tumor suppressor genes.^27,28,29,30,31^ The finding that *BRCA1* methylation was not associated with a family history of breast/ovarian cancer was expected, considering the magnitude of the HRs reported.

Recent trials have provided preliminary evidence suggesting that *BRCA1* methylation may be associated with a more favorable response to chemotherapy, as well as polyadenosine diphosphate-ribose polymerase inhibition, in breast cancer.^8,32^ Neither trial distinguished somatic from constitutional methylation. Thus, it remains to be determined if constitutionally methylated tumors have the same phenotypical characteristics as those methylated somatically.

One may assume that the effect of *BRCA1* methylation and *BRCA1* pathogenic variants is similar in individual cells. In this article, we found the odds ratios for TNBC as well as HGSOC associated with WBC *BRCA1* methylation to be modest as compared with the odds ratio of more than 50 for TNBC in *BRCA1* germline pathogenic variant carriers.^33^ This likely reflects the fact that germline pathogenic variants affect all cells in the body, whereas only a small fraction, 0.1% to 10% of the cells, carry methylated alleles. The present study’s finding that *BRCA1* methylation occurs independent of rs799905 genotype across individuals is consistent with previous findings,^19^ arguing against a cis-acting genetic factor, subject to mendelian inheritance, as the underlying cause of methylation.

An important question is to what extent WBC *BRCA1* methylation represents *BRCA1* methylation in normal tissue across other organs, like the ovaries, fallopian tubes, breasts, and others. Global DNA methylation pattern may vary across different tissue compartments and even between WBC subfractions.^34,35^ However, we previously found WBC *BRCA1* promoter methylation to be strongly associated with methylation status in other benign tissues.^19^ This finding, in concert with the observation that *BRCA1* mosaic promoter methylation occurs across all age groups, including newborns, supports the hypothesis that this methylation is constitutive,^10^ thus affecting different tissue compartments derived from all the embryonic germ layers.

General methylation patterns may change with age.^36^ However, in a previous study,^19^ we found WBC *BRCA1* methylation among females across all age groups, with a slight drop in frequency during lifetime. In the present study, we found *BRCA1* methylation to be predominantly monoallelic, nearly completely restricted to the same allele across affected cells within the same individual, as previously indicated by Hansman and colleagues^13^ in a small group of patients. While this finding does not fully exclude the possibility of dynamic modulations of *BRCA1* methylation through life, it supports the hypothesis that normal tissue *BRCA1* methylation has a clonal origin, arising in a single cell at an early embryonic stage.

An important question is whether *BRCA1* methylation could be a secondary event to germline pathogenic variants in *BRCA1* or other tumor suppressor genes. Our subgroup analysis revealed no evidence for such a covariance. Regarding *BRCA1* pathogenic variants, in a previous study analyzing more than 250 individuals harboring *BRCA1* germline pathogenic variants who had received a diagnosis of HGSOC, we found constitutional methylation frequency to be lower compared with individuals harboring either germline *BRCA2* variants or being wild type for both genes.^19^

### Limitations

All WHI participants were postmenopausal, with a median age of 62 years, with 813 (17%) being  70 years or older at entry. In general, TNBC is more common in younger women. Thus, while TNBC constitutes about 15% of all US breast cancers, the percentage of TNBC in WHI is 7%.^37^ Moreover, the prevalence of tumor *BRCA1* methylation is higher in younger compared with older women with TNBC.^4^ Thus, in our previous study assessing *BRCA1* WBC methylation status in women at all ages who already received a diagnosis of HGSOC^19^ the odds ratio associated with HGSOC varied between 2.2 to 2.9 and was higher among younger compared with older individuals. This indicates that the risk of TNBC and HGSOC associated with WBC *BRCA1* methylation may be underestimated in the present study. While information on *BRCA1* germline pathogenic variant status was not available for all patients in the current study, in the subgroup from whom *BRCA1* methylation could be compared with *BRCA1/2* germline pathogenic variant status, we detected no association between germline variant status and *BRCA1* methylation. Notably, *BRCA1* promoter methylation and *BRCA1* germline pathogenic variants have been reported to be mutually exclusive in breast cancer tissue^4^ as well as in WBC collected from patients with a diagnosis of HGSOC.^19^ The present study was not powered to assess different racial and ethnic groups, precluding conclusions for risk in African American individuals, for whom the incidence of TNBC is known to be elevated.

For cancer risk studies, the findings should be confirmed in independent cohorts. To our knowledge, there are few population-based cohorts that are enrolling sufficient numbers of participants, including follow-ups with regular health assessment, that are adequate to confirm our findings. However, our study contains an indirect independent validation because it suggests that there is an association between *BRCA1* methylation and TNBC and HGSOC, the 2 cancer forms most strongly associated with germline *BRCA1* pathogenic variants.

## Conclusions

In this case-control study, we found normal tissue (WBC) *BRCA1* promoter methylation to be associated with an elevated HR for incident TNBC and HGSOC (also when restricting analysis to cancers developing more than 5 years after WBC sampling). The study findings have 2 major implications. First, WBC *BRCA1* methylation may help identify women at elevated risk of TNBC and HGSOC. This raises the question whether methylation carriers should be offered breast cancer screening at a younger age compared with the general population. While multimodal screening for HGSOC has not been recommended for women without deleterious germline *BRCA1/BRCA2* pathogenic variants,^38^ the study findings add evidence to future discussion of potential screening strategies for this serious cancer diagnosis. Second, the current study findings, in concert with previous data revealing WBC *BRCA1* methylation to occur in women across all age groups, even newborns,^19^ point to *BRCA1* methylation as an early embryonic event that is followed by clonal expansion. Prenatal events have been associated with risk of breast cancer, as well as other cancers.^39,40,41^ Thus, this study’s findings should trigger further research assessing the potential association of constitutional methylation of other genes with the risk of other cancer types and research into the cause(s) of constitutional normal tissue methylation of tumor suppressor genes.

## Notes

Supplement 1. Trial protocol

Supplement 2. eMethods. eResults. eTable 1. GRCh38 genomic coordinates of PCR amplicons and individual CpGs eTable 2. Primer sequences and amplification conditions for PCR amplicons eTable 3. Fractions of methylated samples by case-control status and family history of cancer eTable 4. Fractions of methylated controls by age at screening eTable 5. Fractions of methylated BRCA1 epiallele carriers by hormone therapy group eTable 6. Sample counts in relation to rs799905 SNP genotype. eTable 7. Distribution of germline mutations in shared case and control samples eFigure 1. The genomic structure of the BRCA1 promoter region eFigure 2. Observed versus expected methylation levels in control samples eFigure 3. Empirical cumulative density function (eCDF) plots eFigure 4. Histogram of log10-transformed VEF values for region CpG14–34 eFigure 5. Risk of incident TNBC or HGSOC applying increasing methylation cutoff values eFigure 6. Risk of incident TNBC or HGSOC calculated using optimized cutoffs eFigure 7. Risk of incident TNBC or HGSOC calculated for different sets of CpGs eFigure 8. Risk of incident TNBC or HGSOC calculated for different PCR amplicons eFigure 9. Risk (OR) of incident TNBC or HGSOC calculated using 2x2 contingency tables eFigure 10. Risk (OR) of incident TNBC or HGSOC calculated using multivariate logistic regression eFigure 11. Scatter plot of methylation beta and VEF values eFigure 12. Risk of incident TNBC or HGSOC calculated using beta instead of VEF values eFigure 13. Risk of incident TNBC or HGSOC in subgroups based on hormone therapy exposure eFigure 14. Risk of incident TNBC or HGSOC accounting for potential interaction between methylation and hormone therapy eFigure 15. Risk of incident TNBC or HGSOC calculated for subsets of samples eFigure 16. Allele specificity of methylation eReferences.

## References

[1] Kuchenbaecker KB, Hopper JL, Barnes DR, ; BRCA1 and BRCA2 Cohort Consortium. Risks of breast, ovarian, and contralateral breast cancer for *BRCA1* and *BRCA2* mutation carriers. JAMA. 2017;317(23):2402-2416. doi:10.1001/jama.2017.7112 28632866

[2] Mateo J, Lord CJ, Serra V, . A decade of clinical development of PARP inhibitors in perspective. Ann Oncol. 2019;30(9):1437-1447. doi:10.1093/annonc/mdz192 31218365 PMC6771225

[3] Davies H, Glodzik D, Morganella S, . HRDetect is a predictor of *BRCA1* and *BRCA2* deficiency based on mutational signatures. Nat Med. 2017;23(4):517-525. doi:10.1038/nm.4292 28288110 PMC5833945

[4] Glodzik D, Bosch A, Hartman J, . Comprehensive molecular comparison of BRCA1 hypermethylated and BRCA1 mutated triple negative breast cancers. Nat Commun. 2020;11(1):3747. doi:10.1038/s41467-020-17537-2 32719340 PMC7385112

[5] Konstantinopoulos PA, Ceccaldi R, Shapiro GI, D’Andrea AD. Homologous recombination deficiency: exploiting the fundamental vulnerability of ovarian cancer. Cancer Discov. 2015;5(11):1137-1154. doi:10.1158/2159-8290.CD-15-0714 26463832 PMC4631624

[6] Rice JC, Ozcelik H, Maxeiner P, Andrulis I, Futscher BW. Methylation of the BRCA1 promoter is associated with decreased BRCA1 mRNA levels in clinical breast cancer specimens. Carcinogenesis. 2000;21(9):1761-1765. doi:10.1093/carcin/21.9.1761 10964110

[7] Bell D, Berchuck A, Birrer M, ; Cancer Genome Atlas Research Network. Integrated genomic analyses of ovarian carcinoma. Nature. 2011;474(7353):609-615. doi:10.1038/nature10166 21720365 PMC3163504

[8] Eikesdal HP, Yndestad S, Elzawahry A, . Olaparib monotherapy as primary treatment in unselected triple negative breast cancer. Ann Oncol. 2021;32(2):240-249. doi:10.1016/j.annonc.2020.11.009 33242536

[9] Mazor T, Pankov A, Johnson BE, . DNA methylation and somatic mutations converge on the cell cycle and define similar evolutionary histories in brain tumors. Cancer Cell. 2015;28(3):307-317. doi:10.1016/j.ccell.2015.07.012 26373278 PMC4573399

[10] Lønning PE, Eikesdal HP, Løes IM, Knappskog S. Constitutional mosaic epimutations—a hidden cause of cancer? Cell Stress. 2019;3(4):118-135. doi:10.15698/cst2019.04.183 31225507 PMC6551830

[11] Snell C, Krypuy M, Wong EM, Loughrey MB, Dobrovic A; kConFab investigators. BRCA1 promoter methylation in peripheral blood DNA of mutation negative familial breast cancer patients with a BRCA1 tumour phenotype. Breast Cancer Res. 2008;10(1):R12. doi:10.1186/bcr1858 18269736 PMC2374968

[12] Wong EM, Southey MC, Fox SB, . Constitutional methylation of the BRCA1 promoter is specifically associated with BRCA1 mutation-associated pathology in early-onset breast cancer. Cancer Prev Res (Phila). 2011;4(1):23-33. doi:10.1158/1940-6207.CAPR-10-0212 20978112 PMC4030007

[13] Hansmann T, Pliushch G, Leubner M, . Constitutive promoter methylation of BRCA1 and RAD51C in patients with familial ovarian cancer and early-onset sporadic breast cancer. Hum Mol Genet. 2012;21(21):4669-4679. doi:10.1093/hmg/dds308 22843497 PMC3471399

[14] Iwamoto T, Yamamoto N, Taguchi T, Tamaki Y, Noguchi S. BRCA1 promoter methylation in peripheral blood cells is associated with increased risk of breast cancer with BRCA1 promoter methylation. Breast Cancer Res Treat. 2011;129(1):69-77. doi:10.1007/s10549-010-1188-1 20882403

[15] Bosviel R, Michard E, Lavediaux G, Kwiatkowski F, Bignon YJ, Bernard-Gallon DJ. Peripheral blood DNA methylation detected in the BRCA1 or BRCA2 promoter for sporadic ovarian cancer patients and controls. Clin Chim Acta. 2011;412(15-16):1472-1475. doi:10.1016/j.cca.2011.04.027 21557934

[16] Kontorovich T, Cohen Y, Nir U, Friedman E. Promoter methylation patterns of ATM, ATR, BRCA1, BRCA2 and p53 as putative cancer risk modifiers in Jewish BRCA1/BRCA2 mutation carriers. Breast Cancer Res Treat. 2009;116(1):195-200. doi:10.1007/s10549-008-0121-3 18642075

[17] Azzollini J, Pesenti C, Pizzamiglio S, . Constitutive *BRCA1* promoter hypermethylation can be a predisposing event in isolated early-onset breast cancer. Cancers (Basel). 2019;11(1):E58. doi:10.3390/cancers11010058 30634417 PMC6356733

[18] Prajzendanc K, Domagała P, Hybiak J, . BRCA1 promoter methylation in peripheral blood is associated with the risk of triple-negative breast cancer. Int J Cancer. 2020;146(5):1293-1298. doi:10.1002/ijc.32655 31469414

[19] Lønning PE, Berge EO, Bjørnslett M, . White blood cell *BRCA1* promoter methylation status and ovarian cancer risk. Ann Intern Med. 2018;168(5):326-334. doi:10.7326/M17-0101 29335712

[20] Anderson G, Cummings S, Freedman LS, ; Women’s Health Initiative Study Group. Design of the Women’s Health Initiative clinical trial and observational study. Control Clin Trials. 1998;19(1):61-109. doi:10.1016/S0197-2456(97)00078-0 9492970

[21] Nikolaienko O, Lønning PE, Knappskog S. epialleleR: an R/BioC package for sensitive allele-specific methylation analysis in NGS data. bioRxiv 2022: 2022.06.30.498213. doi:10.1101/2022.06.30.498213 PMC10622323 37919976

[22] Kurian AW, Bernhisel R, Larson K, . Prevalence of pathogenic variants in cancer susceptibility genes among women with postmenopausal breast cancer. JAMA. 2020;323(10):995-997. doi:10.1001/jama.2020.0229 32154851 PMC7064876

[23] Domchek SM, Friebel TM, Singer CF, . Association of risk-reducing surgery in BRCA1 or BRCA2 mutation carriers with cancer risk and mortality. JAMA. 2010;304(9):967-975. doi:10.1001/jama.2010.1237 20810374 PMC2948529

[24] Hitchins MP. Constitutional epimutation as a mechanism for cancer causality and heritability? Nat Rev Cancer. 2015;15(10):625-634. doi:10.1038/nrc4001 26383139

[25] Evans DGR, van Veen EM, Byers HJ, . A dominantly inherited 5′ UTR variant causing methylation-associated silencing of *BRCA1* as a cause of breast and ovarian cancer. Am J Hum Genet. 2018;103(2):213-220. doi:10.1016/j.ajhg.2018.07.002 30075112 PMC6080768

[26] Al-Moghrabi N, Al-Showimi M, Al-Yousef N, . Methylation of BRCA1 and MGMT genes in white blood cells are transmitted from mothers to daughters. Clin Epigenetics. 2018;10(1):99. doi:10.1186/s13148-018-0529-5 30049288 PMC6062990

[27] Steinke-Lange V, de Putter R, Holinski-Feder E, Claes KBM. Somatic mosaics in hereditary tumor predisposition syndromes. Eur J Med Genet. 2021;64(12):104360. doi:10.1016/j.ejmg.2021.104360 34655802

[28] Friedman E, Efrat N, Soussan-Gutman L, . Low-level constitutional mosaicism of a de novoBRCA1 gene mutation. Br J Cancer. 2015;112(4):765-768. doi:10.1038/bjc.2015.14 25633036 PMC4333503

[29] Zhang J, Walsh MF, Wu G, . Germline mutations in predisposition genes in pediatric cancer. N Engl J Med. 2015;373(24):2336-2346. doi:10.1056/NEJMoa1508054 26580448 PMC4734119

[30] Evans DG, Ramsden RT, Shenton A, . Mosaicism in neurofibromatosis type 2: an update of risk based on uni/bilaterality of vestibular schwannoma at presentation and sensitive mutation analysis including multiple ligation-dependent probe amplification. J Med Genet. 2007;44(7):424-428. doi:10.1136/jmg.2006.047753 17307835 PMC2598002

[31] Pareja F, Ptashkin RN, Brown DN, . Cancer-causative mutations occurring in early embryogenesis. Cancer Discov. 2022;12(4):949-957. doi:10.1158/2159-8290.CD-21-1110 34949653 PMC8983494

[32] Stefansson OA, Hilmarsdottir H, Olafsdottir K, . *BRCA1* promoter methylation status in 1031 primary breast cancers predicts favorable outcomes following chemotherapy. JNCI Cancer Spectr. 2019;4(2):pkz100. doi:10.1093/jncics/pkz100 32175521 PMC7061679

[33] Walsh T, Gulsuner S, Lee MK, . Inherited predisposition to breast cancer in the Carolina Breast Cancer Study. NPJ Breast Cancer. 2021;7(1):6. doi:10.1038/s41523-020-00214-4 33479248 PMC7820260

[34] Teschendorff AE, Menon U, Gentry-Maharaj A, . An epigenetic signature in peripheral blood predicts active ovarian cancer. PLoS One. 2009;4(12):e8274. doi:10.1371/journal.pone.0008274 20019873 PMC2793425

[35] Houseman EA, Accomando WP, Koestler DC, . DNA methylation arrays as surrogate measures of cell mixture distribution. BMC Bioinformatics. 2012;13:86. doi:10.1186/1471-2105-13-86 22568884 PMC3532182

[36] Jones MJ, Goodman SJ, Kobor MS. DNA methylation and healthy human aging. Aging Cell. 2015;14(6):924-932. doi:10.1111/acel.12349 25913071 PMC4693469

[37] Chlebowski RT, Aragaki AK, Prentice RL. Dietary moderation and deaths from breast cancer. J Clin Oncol. 2020;38(26):3071-3072. doi:10.1200/JCO.20.01218 32614701

[38] Sroczynski G, Gogollari A, Kuehne F, . A systematic review on cost-effectiveness studies evaluating ovarian cancer early detection and prevention strategies. Cancer Prev Res (Phila). 2020;13(5):429-442. doi:10.1158/1940-6207.CAPR-19-0506 32071120

[39] Denholm R, De Stavola B, Hipwell JH, . Pre-natal exposures and breast tissue composition: findings from a British pre-birth cohort of young women and a systematic review. Breast Cancer Res. 2016;18(1):102. doi:10.1186/s13058-016-0751-z 27729066 PMC5059986

[40] Qiu L, Onoyama S, Low HP, . Effect of preeclampsia on umbilical cord blood stem cells in relation to breast cancer susceptibility in the offspring. Carcinogenesis. 2015;36(1):94-98. doi:10.1093/carcin/bgu231 25398884 PMC4291050

[41] Swerdlow AJ, De Stavola BL, Swanwick MA, Maconochie NES. Risks of breast and testicular cancers in young adult twins in England and Wales: evidence on prenatal and genetic aetiology. Lancet. 1997;350(9093):1723-1728. doi:10.1016/S0140-6736(97)05526-8 9413462

