# Supplementary material for: Constitutional BRCA1 Methylation and Risk of Incident Triple-Negative Breast Cancer and High-grade Serous Ovarian Cancer
Source: JAMA Oncol. 2022 Sep 8;8(11):1579–87. doi: 10.1001/jamaoncol.2022.3846 (PMC9459895; doi:10.1001/jamaoncol.2022.3846)
Supplement: Supplement 1. — Trial protocol [file jamaoncol-e223846-s001.pdf]

November 6, 2018

WHI Ancillary Study #543 Exploring WBC *BRCA1* methylation as a risk factor for incidental cases of high grade serous ovarian cancer and triple-negative breast cancer in WHI

Study population: WHI Clinical Trial and Observational Study Participants (n=161,808)
Cancer outcomes and follow-up data as of 3/31/2018

Specimen requirements: Baseline DNA 1 ug @ 25 ng/ul

Case definition:

a) Invasive triple negative breast cancer (TNBC) defined as estrogen and progesterone receptor negative and HER2 normal status
b) Invasive high grade serous ovarian cancer (HGSOC), includes fallopian tube and peritoneum cancer sites, where serous defined as histology codes 8441/3, 8460/3 and 8461/3, and high-grade defined as either poorly differentiated or anaplastic grade. Cases with grade unknown or not done will also be included.

Exclusions

	TNBC, n=744	HGSOC, n=587
History of breast cancer reported at baseline	48	NA
Bilateral oophorectomy reported at baseline, or missing information	NA	30
Insufficient DNA from baseline blood draw	29	11
Remaining eligible cases	n=668*	N=549*

*Note: there are 5 women who are both a TNBC and HGSOC case. For 4/5 women, the TNBC cancer occurred before the HGSOC, ranging from 7.2 to 12.4 years prior. For 1/5, the HGSOC occurred 6.2 years before the TNBC. These women will be included as both TNBC and HGSOC cases, and matched accordingly. The woman whose HGSOC occurred before the TNBC will be considered to have a history of bilateral oophorectomy in the breast cancer case-control matching.

Characteristics of Eligible Cases

	TNBC, n=668	HGSOC, n=549
Age at blood draw, 5-yr distribution, n (%)		
< 55 yrs	89 (13.3%)	60 (10.9%)
55 – 59 yrs	164 (24.6%)	133 (24.2%)
60 – 64 yrs	161 (24.1%)	149 (27.1%)
65 – 69 yrs	135 (20.2%)	127 (23.1%)
70 – 74 yrs	99 (14.8%)	60 (10.9%)
≥75 yrs	20 (3.0%)	20 (3.6%)
Race/ethnicity, n (%)		
White	547 (81.9%)	505 (92.0%)
Black	90 (13.5%)	19 (3.5%)
Hispanic	10 (1.5%)	16 (2.9%)
American Indian	3 (0.5%)	1 (0.2%)
Asian/PI	14 (1.1%)	4 (0.7%)
Unknown	4 (0.6%)	4 (0.7%)

Bilateral oophorectomy self-reported at baseline		
Unknown	11 (1.7%)	
No	531 (79.5%)	549 (100%)
Yes	126 (18.9%)	
If yes, age at bilateral oophorectomy		
Unknown	1 (0.8%)	
< 30 yrs	2 (1.6%)	
30-34 yrs	5 (4.0%)	
35-39 yrs	13 (10.4%)	
40-44 yrs	25 (20.0%)	
45-49 yrs	33 (26.4%)	
50-54 yrs	26 (20.8%)	
55-59 yrs	9 (7.2%)	
≥ 60 yrs	11 (8.8%)	
WHI Study Component		
Observational study	389 (58.2%)	329 (59.9%)
Clinical Trial	279 (41.8%)	220 (40.1%)
Hormone therapy trial		
Active (estrogen alone or estrogen+progestin)	65 (9.7%)	45 (8.2%)
Placebo	46 (6.9%	26 (4.7%)
Not randomized to trial	557 (83.4%)	478 (87.1%)
Hormone therapy use reported at any time, including at baseline		
Never used	184 (27.5%)	140 (25.5%)
Ever used estrogen alone	217 (32.5%)	143 (26.1%)
Ever used estrogen+progestin	267 (40.0%)	266 (48.4%)
Hormone therapy use prior to diagnosis date		
Never used	192 (28.7%)	151 (27.5%)
Ever used estrogen alone	216 (32.3%)	139 (25.3%)
Ever used estrogen+progestin	260 (38.9%)	259 (47.2%)
DNA Extraction Method, n (%)		
No extracted DNA	451 (67.5%)	413 (75.2%)
Extracted using Qiagen/Five Prime	153 (22.8%)	119 (21.7%)
Extracted using Phenol Chloroform	39 (5.8%)	13 (2.4%)
Extracted using Salt-precipitation	22 (3.3%)	3 (0.6%)
Extracted using Qiagen/Bioserve	3 (0.5%)	1 (0.2%)

Control sample:

Eligible controls are drawn from the CT+OS after excluding the TNBC and HGSOC cases

Controls were excluded for the following reasons:
a) History of breast or ovarian cancer reported at baseline or at any time during follow-up
b) No follow-up
c) No DNA
d) History of bilateral oophorectomy for HGSOC controls only

Number of eligible controls: n=139,057 (when d applied: n=107,353)

Case-control matching (TNBC 1:3, HGSOC 1:6)
Matching criteria:
a) Age (± 3 years)
b) Race/ethnicity, exact match
c) DNA extraction method, exact match (if no extracted DNA, method= Qiagen/Five Prime)
d) Alive and free of breast or ovarian cancer at time of index case diagnosis
e) Hormone therapy use categorized as never/ever estrogen alone/ever estrogen+progestin, where estrogen+progestin use takes precedence over estrogen alone use (HT_type coded as 0=never, 1=ever Ealone, 2=ever E+P). For cases, HT_type is defined during time interval prior to diagnosis date, which includes use reported at baseline. Controls are matched such that they had matching HT_type at any time prior to the index case diagnosis date.
f) For TNBC only, bilateral oophorectomy (yes, no or unknown), and if yes, matched on age at oophorectomy, where age at oophorectomy is categorized as: < 40, 40-44, 45-49, ≥50.

Control selection will be done in a time-forward manner, selecting controls for each case from the risk set at the time of the case's event. The matching algorithm will be allowed to select the closest match based on a criteria to minimize an overall distance measure (Bergstralh EJ, Kosanke JL. Computerized matching of cases to controls. Technical Report #56, Department of Health Sciences Research, Mayo Clinic, Rochester MN. April 1995). Each matching factor will be given the same weight. We will try to utilize as many controls as possible in a double manner, i.e. matched to both a TNBC and HGSOC case, however a case cannot be a control for another case.

Matching results

I. HGSOC cases were matched first, N=549 cases matched to 6 controls per case

A. Never used HT prior to dx, N=151
151 matched to 6 controls
Total # controls = 906

B. Used HT prior to dx, N= 397 (139 Ealone, 259 E+P)
397 matched to 6 controls
1 matched to 5 controls *(incomplete matching)*
Total # controls = 2387

A+B combined: total # controls = 3293*
548 cases matched to 6 controls
1 case matched to 5 controls *(incomplete matching)*

II. TNBC cases were matched next, N=668 cases matched to 3 controls per case

A. No bilateral oophorectomy and never used HT prior to dx, N=175

Step 1. Using HGSOC controls
133 matched to 3 HGSOC controls
2 matched to 2 HGSOC controls*
5 matched to 1 HGSOC control**
35 matched to 0 HGSOC controls***

Step 2. Using remaining control pool
*2 matched to 1 control
**5 matched to 2 controls
***35 matched to 3 controls

B. No bilateral oophorectomy and used HT prior to dx, N=356 (119 Ealone, 237 E+P)

Step 1. Using HGSOC controls
311 matched to 3 HGSOC controls
4 matched to 2 HGSOC controls*
10 matched to 1 HGSOC control**
31 matched to 0 HGSOC controls***

Step 2. Using remaining control pool
*4 matched to 1 control
**10 matched to 2 controls
***28 matched to 3 controls; *1 matched to 2 controls, 2 matched to 1 control; no additional controls available (incomplete matching)*

C. Bilateral oophorectomy and never used HT prior to dx, N=16
14 matched to 3 controls
1 matched to 2 controls; *no additional controls available (incomplete matching)*
1 matched to 1 control; *no additional controls available (incomplete matching)*

D. Bilateral oophorectomy and used HT prior to dx, N=110 (87 Ealone, 23 E+P)
108 matched to 3 controls
2 matched to 2 controls; *no additional controls available (incomplete matching)*

E. Unknown bilateral oophorectomy and never used HT prior to dx, N=1
1 matched to 3 controls

F. Unknown bilateral oophorectomy and used HT prior to dx, N=10 (10 Ealone, 0 E+P)
10 matched to 3 controls

A-F combined: total # controls = 1994 (1359 also matched to a HGSOC case)
661 cases matched to 3 controls
4 cases matched to 2 controls *(incomplete matching)*
3 case matched to 1 control *(incomplete matching)*

Final Matching Summary

Total number of cases with complete matching: 548 + 661 = 1209 *
Total number of cases with incomplete matching: 1 + 7 = 8
Total number of matched controls: 3293 + 1994 – 1359 = 3928

*the 5 women with both TNBC and HGSOC are counted twice in this count of cases.

Tumor Characteristics of Selected Cases

	HGSOC, n=549
Behavior	
Invasive	549 (100%)
Histology	
8441 – Serous cystadenocarcinoma, NOS	268 (48.8%)
8460 – Papillary cystadenocarcinoma, NOS	269 (49.0%)
8461 – Serous surface papillary carcinoma	11 (2.0%)
8441/8460*	1 (0.2%)
Grade	
Poorly differentiated	227 (41.3%)
Anaplastic	178 (32.4%)
Unknown/not done	144 (26.2%)
Site	
Ovary	410 (74.7%)
Fallopian tube	55 (10.0%)
Peritoneum	83 (15.1%)
Fallopian tube and peritoneum*	1 (0.2%)

*one case was diagnosed with both fallopian tube and peritoneum cancer on the same date; the grade is the same for both sites, but the histology code differs, where 8441 is for the fallopian tube and 8460 the peritoneum.

	TNBC, n=668
Behavior	
Invasive	668 (100%)
Histology	
8010 – Carcinoma, NOS	4 (0.6%)
8032 – Spindle cell carcinoma	10 (1.5%)
8033 – Pseudosarcomatous carcinoma	1 (0.2%)
8041 – Small cell carcinoma nos	1 (0.2%)
8070 – Squamous cell carcinoma nos	2 (0.3%)
8090 – Basal cell carcinoma, nos	1 (0.2%)
8123 – Basaloid carcinoma	1 (0.2%)
8140 – Adenocarcinoma nos	3 (0.5%)
8200 – Adenoid cystic carcinoma	3 (0.5%)
8201 – Cribriform carcinoma	1 (0.2%)
8211 – Tubular adenocarcinoma	2 (0.3%)
8246 – Neuroendocrine carcinoma	2 (0.3%)
8401 – Apocrine adenocarcinoma	23 (3.4%)
8480 – Mucous adenocarcinoma	4 (0.6%)

8500 – Infiltrating duct carcinoma	526 (78.7%)
8501 – Comedocarcinoma, nos	1 (0.2%)
8502 – Juvenile carcinoma, breast	1 (0.2%)
8503 – Intraductal adenocarcinoma, papillary, with invasion	2 (0.3%)
8510 – Medullary carcinoma, nos	12 (1.8%)
8520 – Lobular carcinoma nos	19 (2.8%)
8522 – Infiltrating duct and lobular carcinoma	32 (4.8%)
8530 – Inflammatory carcinoma	4 (0.6%)
8541 – Paget disease and infiltrating duct carcinoma of breast	2 (0.3%)
8560 – Adenosquamous carcinoma	1 (0.2%)
8562 – Epithelial-myoepithelial carcinoma	1 (0.2%)
8570 – Adenocarcinoma with squamous metaplasia	4 (0.6%)
8572 – Adenocarcinoma with spindle cell metaplasia	2 (0.3%)
8802 – Giant cell sarcoma	1 (0.2%)
8980 – Carcinosarcoma, nos	1 (0.2%)
9220 – Chondrosarcoma nos	1 (0.2%)
Grade	
Well differentiated	29 (4.3%)
Moderately differentiated	12 (21.3%)
Poorly differentiated	443 (66.3%)
Anaplastic	28 (4.2%)
Unknown/not done	26 (3.9%)
