# Supplementary material for: Constitutional BRCA1 Methylation and Risk of Incident Triple-Negative Breast Cancer and High-grade Serous Ovarian Cancer
Source: JAMA Oncol. 2022 Sep 8;8(11):1579–87. doi: 10.1001/jamaoncol.2022.3846 (PMC9459895; doi:10.1001/jamaoncol.2022.3846)
Supplement: Supplement 2. — eMethods. eResults. eTable 1. GRCh38 genomic coordinates of PCR amplicons and individual CpGs eTable 2. Primer sequences and amplification conditions for PCR amplicons eTable 3. Fractions of methylated samples by case-control status and family history of cancer eTable 4. Fractions of methylated controls by age at screening eTable 5. Fractions of methylated BRCA1 epiallele carriers by hormone therapy group eTable 6. Sample counts in relation to rs799905 SNP genotype. eTable 7. Distribution of germline mutations in shared case and control samples eFigure 1. The genomic structure of the BRCA1 promoter region eFigure 2. Observed versus expected methylation levels in control samples eFigure 3. Empirical cumulative density function (eCDF) plots eFigure 4. Histogram of log10-transformed VEF values for region CpG14–34 eFigure 5. Risk of incident TNBC or HGSOC applying increasing methylation cutoff values eFigure 6. Risk of incident TNBC or HGSOC calculated using optimized cutoffs eFigure 7. Risk of incident TNBC or HGSOC calculated for different sets of CpGs eFigure 8. Risk of incident TNBC or HGSOC calculated for different PCR amplicons eFigure 9. Risk (OR) of incident TNBC or HGSOC calculated using 2x2 contingency tables eFigure 10. Risk (OR) of incident TNBC or HGSOC calculated using multivariate logistic regression eFigure 11. Scatter plot of methylation beta and VEF values eFigure 12. Risk of incident TNBC or HGSOC calculated using beta instead of VEF values eFigure 13. Risk of incident TNBC or HGSOC in subgroups based on hormone therapy exposure eFigure 14. Risk of incident TNBC or HGSOC accounting for potential interaction between methylation and hormone therapy eFigure 15. Risk of incident TNBC or HGSOC calculated for subsets of samples eFigure 16. Allele specificity of methylation eReferences. [file jamaoncol-e223846-s002.pdf]

Supplemental Online Content

Lønning PE, Nikolaienko O, Pan K, et al. Constitutional *BRCA1* methylation and risk of incident triple-negative breast cancer and high-grade serous ovarian cancer. *JAMA Oncol.* Published online September 8, 2022. doi:10.1001/jamaoncol.2022.3846

eMethods.
eResults.
eTable 1. GRCh38 genomic coordinates of PCR amplicons and individual CpGs
eTable 2. Primer sequences and amplification conditions for PCR amplicons
eTable 3. Fractions of methylated samples by case-control status and family history of cancer
eTable 4. Fractions of methylated controls by age at screening
eTable 5. Fractions of methylated *BRCA1* epiallele carriers by hormone therapy group
eTable 6. Sample counts in relation to rs799905 SNP genotype.
eTable 7. Distribution of germline mutations in shared case and control samples
eFigure 1. The genomic structure of the *BRCA1* promoter region
eFigure 2. Observed versus expected methylation levels in control samples
eFigure 3. Empirical cumulative density function (eCDF) plots
eFigure 4. Histogram of log10-transformed VEF values for region CpG14–34
eFigure 5. Risk of incident TNBC or HGSOC applying increasing methylation cutoff values
eFigure 6. Risk of incident TNBC or HGSOC calculated using optimized cutoffs
eFigure 7. Risk of incident TNBC or HGSOC calculated for different sets of CpGs
eFigure 8. Risk of incident TNBC or HGSOC calculated for different PCR amplicons
eFigure 9. Risk (OR) of incident TNBC or HGSOC calculated using 2x2 contingency tables
eFigure 10. Risk (OR) of incident TNBC or HGSOC calculated using multivariate logistic regression
eFigure 11. Scatter plot of methylation beta and VEF values
eFigure 12. Risk of incident TNBC or HGSOC calculated using beta instead of VEF values
eFigure 13. Risk of incident TNBC or HGSOC in subgroups based on hormone therapy exposure
eFigure 14. Risk of incident TNBC or HGSOC accounting for potential interaction between methylation and hormone therapy
eFigure 15. Risk of incident TNBC or HGSOC calculated for subsets of samples
eFigure 16. Allele specificity of methylation
eReferences.

This supplemental material has been provided by the authors to give readers additional information about their work.

© 2022 Lønning PE et al. *JAMA Oncology*.

eMethods

Statistical power estimates

A detailed description of estimates for statistical power is given in the attached study protocol. In brief, power estimates were based on previous results of methylation frequency among patients diagnosed with high-grade serous ovarian cancer (HGSOC) and healthy controls.[1] Given that we did not have information about *BRCA1* germline mutation status among WHI participants (which have been shown mutually exclusive to tumor *BRCA1* methylation), we calculated the power estimates under different scenarios, including a "worst case scenario", assuming a 15% loss of power due to a potential dilution factors like *BRCA1* germline mutations. Assuming a hypermethylation frequency of 4% among healthy individuals and an hazard ratio (HR) of 2, matching 600 triple-negative breast cancers (TNBC) on a 1:3 basis with 1,800 control samples in a nested design provides a power (1-β) of 0.88. As for HGSOC, comparing n=400 patients on a 1:6 basis with 2,400 controls provides a 1-β of 0.80. As such, knowing the availability of >600 TNBC cases and >500 HGSOC cases in the WHI biobank, we found 1:3 and 1:6 nested designs, respectively, to provide adequate power to test the hypothesis.

Selection of cases and case-controls matching

Cases of triple-negative breast cancer (TNBC) and high-grade serous ovarian cancer (HGSOC) were selected from the Women's Health Initiative (WHI) Clinical Trial and Observational Study Participants (n=161,808) according to follow-up data as of 31/3/2018. All details on included subdiagnoses and matching of controls are given in **Supplement 1**.

In brief, a formal selection of n=668 TNBC and n=549 HGSOC cases was made, together with n=3,928 controls, out of which a fraction served as controls versus both TNBC and HGSOC (see below). Subsequent to selection, samples with too low DNA concentration were removed together with those matched controls that were selected on the basis of being matched to cases with too low DNA concentration. Thus, the final sample set consisted of 4,692 samples, out of which 637 were TNBC cases, 511 were HGSOC cases and 3,549 were controls (5 samples were from individuals with both a TNBC and a HGSOC diagnosis). Out of the 3,549 controls, 1,272 served as controls for both TNBC and HGSOC (see Figure 1 in the main text).

WHI DNA Storage, Processing and QA

Entry blood samples were obtained after at least 12 hours of fasting via a prespecified protocol standardized across all study sites. Blood samples were processed, buffy coat aliquoted and stored in freezers at −70°C within 2 hours of collection and shipped on dry ice to a central processing facility (Fisher Bioservices) where storage at −80°C was maintained.

To maintain consistency in handling, all specimen processing (DNA extraction and specimen aliquoting) occurs in the central WHI lab (Fred Hutch Specimen Processing Lab). Since 2008, the WHI DNA extraction procedure is the manual 5-Prime procedure. Prior to 2008, WHI used 3 other methods of extraction: Bioserve, salt precipitation, and phenol/chloroform. 85% of the samples in this study were extracted using the 5-Prime method. DNA was quantified by fluorescence (PicoGreen). For the samples extracted early in the study (prior to 2007), DNA concentration was measured spectrophotometrically with the 260/280 OD ratio.

To ensure that sufficient sample arrives in the testing lab, WHI does not generally distribute aliquots that are < 1 ug and does not generally dilute DNA to a concentration less than 50 ng/ul. Some DNA samples are less than 50 ng/ul upon extraction. They were provided as-is if they met the testing lab's minimum requirements. If a sample was quantitated using the OD ratio, twice as much will be provided to ensure there is enough DNA.

All studies requesting DNA samples are required to include blind duplicates as quality control samples: 5% of the total number of participant samples (2.5% blind duplicate pairs). The study investigator is required to report to the WHI all duplicates identified. From this list, the WHI will confirm the correct/incorrect identification of the blind duplicates and any unexpected duplicates.

Human HCT116 DKO Non-Methylated and Methylated DNA control samples (Zymo Research, cat.no. D5014-1 and D5014-2 respectively) and their mixes with varying ratios were used to test assay sensitivity.

Region of the *BRCA1* promoter selected for analyses and assay design

The genomic structure of the *BRCA1* promoter region is shown in the eFigure 1. The assay applied in the present study covered 50 CpGs (termed CpG00–CpG49). The genomic coordinates for the individual CpGs are given in the eTable 1.

© 2022 Lønning PE et al. *JAMA Oncology*.

eFigure 1. The genomic structure of the *BRCA1* promoter region.

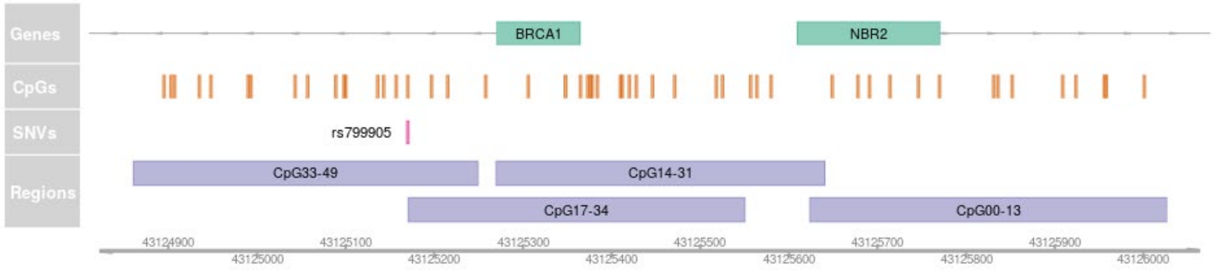

The first exons of the BRCA1 and NBR2 genes are depicted by green rectangles, covered CpGs are indicated by orange vertical lines, rs799905 single-nucleotide variation by pink vertical line, and the four sequenced amplicons by blue rectangles.

eTable 1. GRCh38 genomic coordinates of PCR amplicons and individual CpGs

Type	Name	Coordinates
amplicon	CpG00–13	chr17:43125624–43126026
amplicon	CpG14–31	chr17:43125270–43125640
amplicon	CpG17–34	chr17:43125171–43125550
amplicon	CpG33–49	chr17:43124861–43125249
CpG	CpG00	chr17:43126000–43126001
CpG	CpG01	chr17:43125957–43125958
CpG	CpG02	chr17:43125955–43125956
CpG	CpG03	chr17:43125923–43125924
CpG	CpG04	chr17:43125908–43125909
CpG	CpG05	chr17:43125851–43125852
CpG	CpG06	chr17:43125835–43125836
CpG	CpG07	chr17:43125830–43125831
CpG	CpG08	chr17:43125769–43125770
CpG	CpG09	chr17:43125745–43125746
CpG	CpG10	chr17:43125713–43125714
CpG	CpG11	chr17:43125690–43125691
CpG	CpG12	chr17:43125677–43125678
CpG	CpG13	chr17:43125648–43125649
CpG	CpG14	chr17:43125579–43125580
CpG	CpG15	chr17:43125563–43125564
CpG	CpG16	chr17:43125556–43125557
CpG	CpG17	chr17:43125524–43125525
CpG	CpG18	chr17:43125517–43125518
CpG	CpG19	chr17:43125470–43125471
CpG	CpG20	chr17:43125445–43125446
CpG	CpG21	chr17:43125427–43125428
CpG	CpG22	chr17:43125419–43125420
CpG	CpG23	chr17:43125411–43125412

Type	Name	Coordinates
CpG	CpG23	chr17:43125411–43125412
CpG	CpG24	chr17:43125409–43125410
CpG	CpG25	chr17:43125383–43125384
CpG	CpG26	chr17:43125377–43125378
CpG	CpG27	chr17:43125375–43125376
CpG	CpG28	chr17:43125372–43125373
CpG	CpG29	chr17:43125364–43125365
CpG	CpG30	chr17:43125347–43125348
CpG	CpG31	chr17:43125305–43125306
CpG	CpG32	chr17:43125257–43125258
CpG	CpG33	chr17:43125214–43125215
CpG	CpG34	chr17:43125196–43125197
SNP	SNP	chr17:43125169–43125170
CpG	CpG35	chr17:43125156–43125157
CpG	CpG36	chr17:43125142–43125143
CpG	CpG37	chr17:43125135–43125136
CpG	CpG38	chr17:43125099–43125100
CpG	CpG39	chr17:43125097–43125098
CpG	CpG40	chr17:43125088–43125089
CpG	CpG41	chr17:43125056–43125057
CpG	CpG42	chr17:43125042–43125043
CpG	CpG43	chr17:43124992–43124993
CpG	CpG44	chr17:43124989–43124990
CpG	CpG45	chr17:43124947–43124948
CpG	CpG46	chr17:43124934–43124935
CpG	CpG47	chr17:43124906–43124907
CpG	CpG48	chr17:43124902–43124903
CpG	CpG49	chr17:43124894–43124895

© 2022 Lønning PE et al. *JAMA Oncology*.

Library preparation and sequencing

For each sample, 500 ng of genomic DNA was bisulfite converted using EZ-96 DNA Methylation-Lightning™ Kit (Zymo Research, cat.no. D5033) according to manufacturer's instructions. Converted DNA was used as a template for *BRCA1* gene promoter fragment amplification using KAPA HiFi HotStart Uracil+ ReadyMix PCR Kit (Roche, cat.no. KK2802) and four pairs of primers that do not overlap with any of the CpG dinucleotides (eTable 2). Betaine was added to every PCR mix at a final concentration of 0.5 M to enhance amplification of GC-rich sequences and thus decrease potential PCR bias towards unmethylated sequences. The following thermal cycling conditions were used: 95 °C for 5 min, then 35–47 cycles of 98 °C for 20 s, 56–62 °C for 15–20 s, and 72 °C for 15 s, before a final elongation step of 72 °C for 1 min (details in the eTable 2). All four amplification reactions were pooled per sample and the amplicons were purified using Mag-Bind® TotalPure NGS Kit (Omega Bio-Tek, cat.no. M1378-01) with a bead ratio of 0.65x. Illumina indexes were added to the amplicon mix using Swift Accel-NGS® Methyl-Seq Dual Indexing Kit (Swift Biosciences, cat.no. 38096) and KAPA HiFi HotStart Uracil+ ReadyMix PCR Kit (Roche, cat.no. KK2802) with the following thermal cycling conditions: 98 °C for 45 s, then 6 cycles of 98 °C for 15 s, 66 °C for 30 s, and 72 °C for 30 s, before a final elongation step of 72 °C for 1 min. Indexed libraries were again purified using Mag-Bind® Total Pure NGS Kit (Omega Bio-Tek, cat.no. M1378-01) with a bead ratio of 0.65x and quantified using Quant-iT™ PicoGreen™ dsDNA Assay Kit (ThermoFisher Scientific, cat.no. P11496). Indexed libraries from 96 samples were pooled together and sequenced using Illumina MiSeq Reagent Kit v3 (Illumina, cat.no. MS-102-3003) on an Illumina MiSeq System (Illumina), aiming to achieve an ultra-deep coverage of more than 20,000x for each amplicon (by 2x226 bp long paired end sequencing reads).

eTable 2. Primer sequences and amplification conditions for PCR amplicons

amplicon	forward primer index	forward primer sequence	reverse primer index	reverse primer sequence	PCR template input	annealing temp.	annealing time	PCR cycles	PCR product length
CpG00–13	P5	GATCTACACTCTTTCCCTACACGACGCTCTTCCGATCTaggtttagtttttgtttttaa	P7	GTGACTGGAGTTCAGACGTGTGCTCTTCCGATCTctctaacctctactcttcca	100 ng	56 °C	20 sec	37	475 bp
CpG14–31	P7	GTGACTGGAGTTCAGACGTGTGCTCTTCCGATCTaagagtagaggttagagggtaggt	P5	GATCTACACTCTTTCCCTACACGACGCTCTTCCGATCTcctttacccaaaacaaaaaata	100 ng	56 °C	20 sec	35	443 bp
CpG17–34	P5	GATCTACACTCTTTCCCTACACGACGCTCTTCCGATCTagattgggtggttaatttagagt	P7	GTGACTGGAGTTCAGACGTGTGCTCTTCCGATCTtcaaaaaataccatctatcaac	100 ng	58 °C	20 sec	35	452 bp
CpG33–49	P7	GTGACTGGAGTTCAGACGTGTGCTCTTCCGATCTgatagggggtttaagtgatgtt	P5	GATCTACACTCTTTCCCTACACGACGCTCTTCCGATCTattctcctacctcaacctccta	200 ng	62 °C	15 sec	35	461 bp

Mapping, methylation calling and statistical analysis

Reads were mapped/aligned to the GRCh38 reference genome and the methylation was called using Illumina DRAGEN Bio-IT Platform (v3.6.3) with the following settings: --Aligner.aln-min-score -1000000, --Aligner.min-score-coeff 0, --Aligner.match-score 1, --Aligner.pe-stat-mean-insert 385, --Aligner.pe-stat-stddev-insert 20.0, --Aligner.pe-stat-mean-read-len 220, --Aligner.pe-stat-quartiles-insert "355 385 420". R software environment for statistical computing (v4.0.3) was used for all downstream statistical analyses. Calling hypermethylated variant epiallele frequencies (VEFs) was performed using epialleleR R package (https://bioconductor.org/packages/epialleleR/, v0.99.3)[2] with the following parameters: min.mapq=30, threshold.reads=TRUE, threshold.context="CG", min.context.sites=2, min.context.beta=0.5, max.outofcontext.beta=0.1. Allele frequencies of SNP rs799905 in methylated and unmethylated reads were determined using function epialleleR::generateVcfReport with min.baseq parameter set to 13 to reduce the effect of sequencing errors. Optimization of cutoff values was performed using R package OptimalCutpoints (v1.1-4),[3] using average population prevalences of 0.015 and 0.006 for TNBC and HGSOC, respectively, to adjust for enrichment of cases in the present sample set. Unless stated otherwise, hazard ratio (HR) estimation was performed using Cox proportional hazards regression in matching case-control groups, with the model including age, race/ethnicity, previous hormone use, oophorectomy (for TNBC only) and DNA extraction method as independent variables (covariates).

© 2022 Lønning PE et al. *JAMA Oncology*.

eResults

Allelic distribution of methylation

As neighboring CpGs in the human genome often show concordant methylation,[4] we hypothesized that monoallelic inactivation by methylation will lead to the presence of hypermethylated reads at varying frequencies. Assessing frequencies of hypermethylated epialleles instead of standard beta values is expected to provide enhanced sensitivity and specificity by eliminating the effect of spontaneous deamination events,[5] sequencing errors, potentially spurious single-base methylation events, as well as batch effects due to varying bisulfite conversion efficiency.

To confirm the presence of hypermethylated reads and assess the potential amplification bias, we compared expected and observed hypermethylated variant epiallele frequencies (VEF) for pure samples and dilution series of human HCT116 DKO Non-Methylated and Methylated control DNA, ranging from 100:1 to 1:1). The results showed a moderate preference for amplification of hypomethylated epialleles (PCR bias), although the theoretical and observed VEF values for hypermethylated epialleles were in very good agreement (b=0.7377, standard error of 0.0239, as described in,[6,7] versus beta values' b of 0.6456, standard error of 0.0813; eFigure 2). Thus, no PCR bias correction was performed prior the analysis.

eFigure 2. Observed versus expected methylation levels in control samples

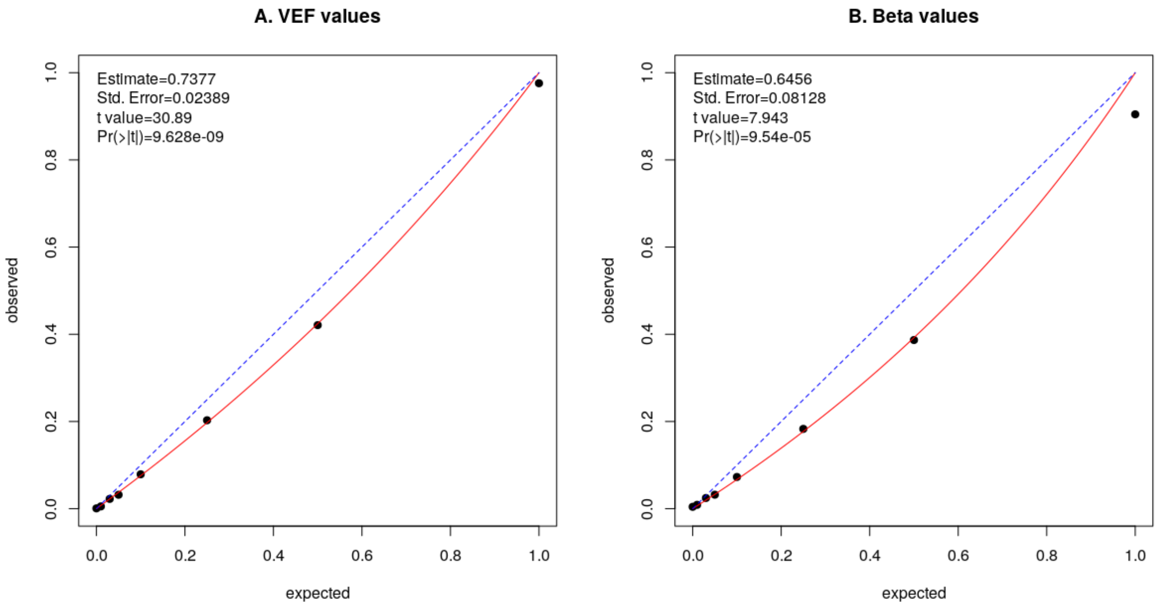

Observed versus expected methylation levels in a dilution series of fully methylated DNA in unmethylated DNA. Methylation calculated as A) VEF and B) beta values. Dotted blue line indicates optimal concordance between observed and expected results. Black dots (with solid red trend line) indicate the results from the analysed dilution series.

Additionally, we assessed the cumulative distribution of average per-read beta values (as an example, empirical cumulative density function (eCDF) plots for 1:1 control DNA mix and a representative real sample are given in the eFigure 3). The results suggest that, although PCR amplification introduces moderate amounts of chimeric reads (reads with mixed CpG methylation and corresponding average beta values within [0.25, 0.75]), the majority of reads derive from either unmethylated or fully methylated templates. Moreover, the absence of reads with average beta value of 0.5 or higher in non-CpG context suggests high bisulfite conversion efficiency, as further confirmed by the average CHH methylation of 1.25% across the entire study. Similar conclusions were drawn from other controls.

In experimental samples, in general, the level of methylation was in the lower range of the control dilution series. Importantly, there was high level of similarity to the control samples, in the sense that the fraction of reads with mixed CpG methylation was very low and the vast majority of reads derived from either unmethylated or fully methylated templates (eFigure 3). This strongly indicates that few cells (if any) have epialleles with a mix of methylated and unmethylated cytosines in CpG context, especially within the core CpG14–34 area, used for main analyses in the present study.

© 2022 Lønning PE et al. *JAMA Oncology*.

eFigure 3. Empirical cumulative density function (eCDF) plots

A

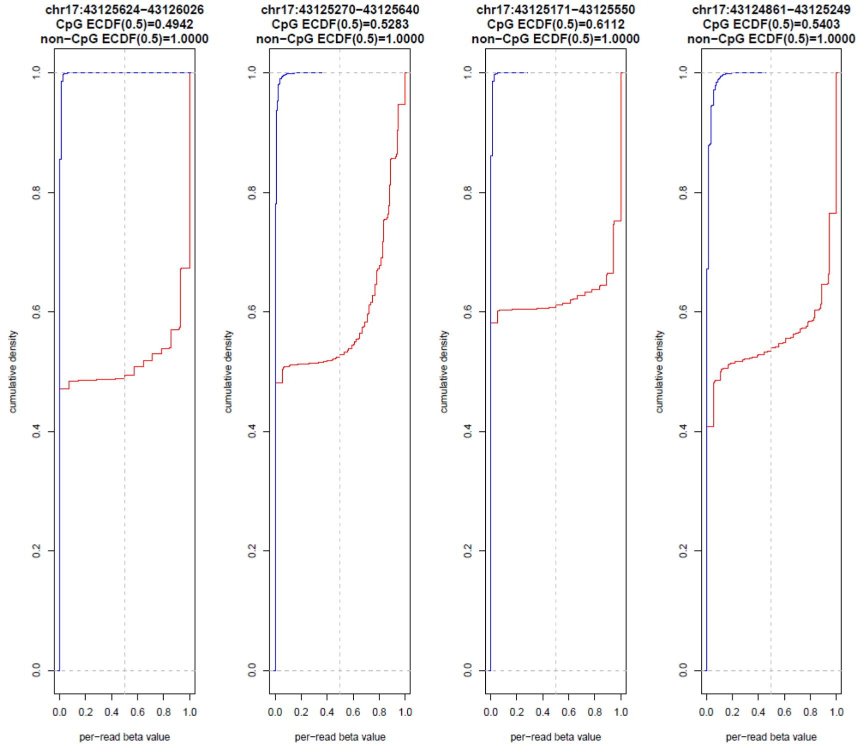

B

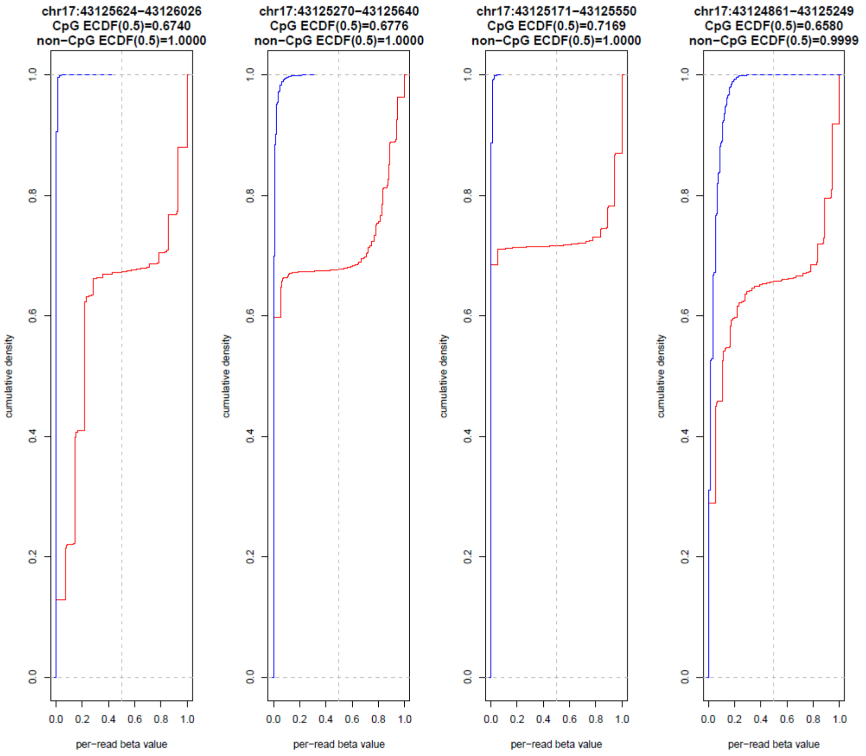

Empirical cumulative density function (eCDF) plots of average per-read beta values across all four amplicons for (A) the control sample consisting of methylated and unmethylated DNA mixed with the ratio of 1:1, and (B) the sample from the individual with the highest VEF (0.2960) across the study. Red lines represent cytosines in CpG context. Blue lines represent cytosines in non-CpG context. Numeric values of “CpG eCDF(0.5)” and “non-CpG eCDF(0.5)” on top of each panel represent the VEF of unmethylated epiallele in CpG and non-CpG contexts, respectively.

© 2022 Lønning PE et al. *JAMA Oncology*.

Estimation of assay sensitivity, specificity, positivity cutoffs and potential experimental biases

Of four amplicons covering *BRCA1* promoter region, amplicons CpG14–31 and CpG17–34 overlap with the region where DNA methylation was found to be associated with the risk of TNBC and HGSOC as described by us and others previously.[1,8] Therefore, and in accordance with the Study Protocol, the average frequency of hypermethylated epialleles covered by amplicons CpG14–31 and CpG17–34 was used as a main metric to assess *BRCA1* promoter methylation (further referred to as region CpG14–34). As anticipated, compared to genomic region-averaged beta values, this combined VEF metric had lower minimum values as well as wider range of detected values ([0, 0.2960] with nonzero values varying within ~5 orders of magnitude, versus beta values' [0.0021, 0.2781] and ~2 orders of magnitude).

In addition to case/control samples and methylation control samples described above, the extended set of samples analysed contained ~5% (133 pairs) of blind duplicates to ensure assay reproducibility. The results for blind duplicates confirmed high robustness and reproducibility of experimental procedures (Pearson's r=0.9437 for CpG14–34 VEF). Thereafter, blind duplicates were excluded from all subsequent analyses.

Under ideal conditions, the sensitivity of an NGS-based assay is limited by the sequencing depth (this study's median coverage of 26500) and by the frequency of the least common allele (~1/31000 for 100 ng of female genomic DNA input). However, the bisulfite conversion reaction is known to heavily affect DNA quality, reducing the number of intact molecules to less than 4%,[9] and thus limiting the maximum theoretical assay sensitivity to approximately 1/1240 or 8.1e-04.

The index switching during multiplex library sequencing has been reported for the patterned as well as non-patterned flow cells.[10,11] With the maximum reported index swap rate for Illumina MiSeq System of 0.67% (although, typically lower for PCR-plus approaches) and maximum fraction of fully methylated reads per library pool of 2%, the frequency of misassigned fully methylated reads is estimated to be no more than 1.4e-04, which is below the maximum theoretical assay sensitivity. In agreement with this, no signs of cross-contamination for samples directly adjacent to fully methylated control sample was observed (average VEF of hypermethylated epiallele of 5.0e-05).

Positivity cutoff value for experimental samples (equals **3.76e-04** for the region CpG14–34) was computationally defined as VEF value with the lowest probability in the range [ISR, MTS] (where ISR is index swap rate of 1.4e-04, and MTS—maximum theoretical sensitivity of 8.1e-04, as calculated above; eFigure 4). **As per protocol, this definition was done and the cutoff was determined based on the entire data set, before unblinding to the case-controls status of the individual samples**.

eFigure 4. Histogram of log10-transformed VEF values for region CpG14–34

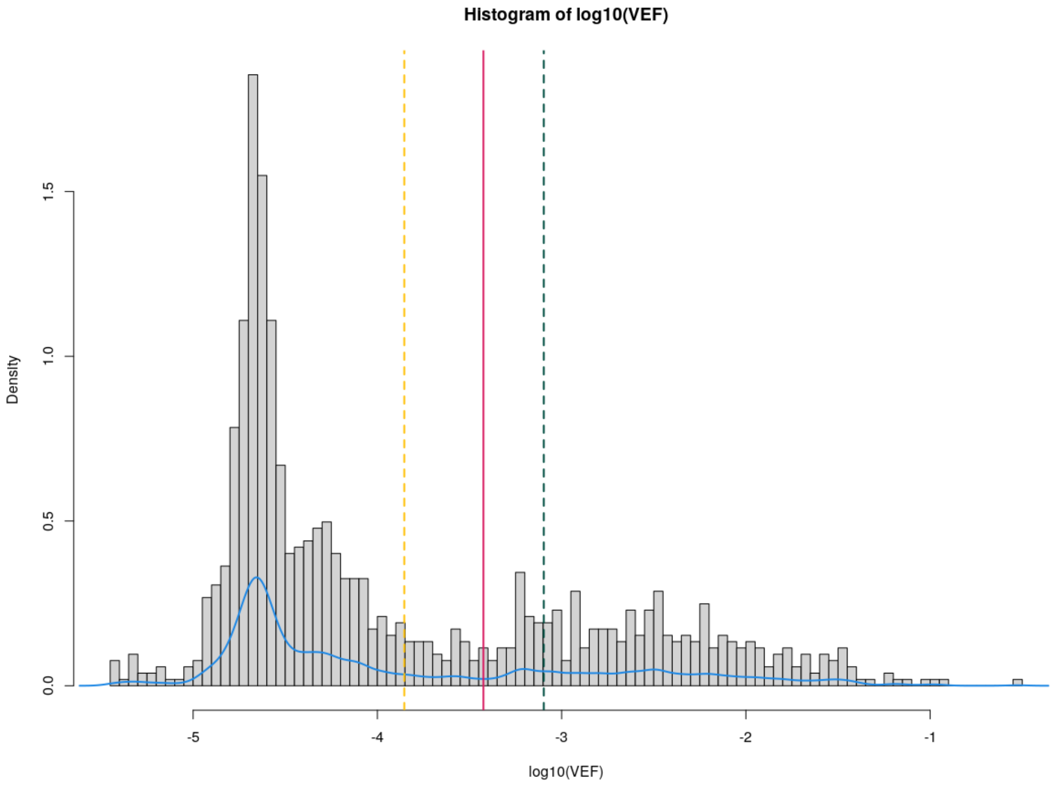

Histogram of log10-transformed VEF values for region CpG14–34. Density is based on binned values from all samples (cases and controls) analyzed in the present study. The blue solid line represents the probability density function. Vertical lines represent maximum index swap rate (dashed yellow), maximum theoretical sensitivity (dashed green), and the optimal cutoff value (solid pink). The latter was determined blinded to case-control status of individual samples and used as cutoff for all main analyses in the present study.

© 2022 Lønning PE et al. *JAMA Oncology*.

Fractions of methylated samples according to family history of cancer

Using the available data on family history of cancer we calculated fractions of individuals carrying WBC *BRCA1* methylation in each category. We did not observe significant differences in methylation frequency between individuals with and without family history of breast or ovarian cancer (eTable 3).

eTable 3. Fractions of methylated samples by case-control status and family history of cancer

		TNBC cases		HGSOC cases		Controls	
		**unmethylated**	**methylated**	**unmethylated**	**methylated**	**unmethylated**	**methylated**
**Family history of breast cancer**	No	137	20	121	15	950	56
	Yes	137	17	85	9	569	30
	Missing	283	42	255	24	1832	108
**Family history of ovarian cancer**	No	253	35	188	20	1373	78
	Yes	16	2	12	1	72	3
	Don't know	16	0	14	3	104	6
	Missing	272	42	247	24	1802	107

Fractions of methylated controls according to age group

Previous studies have found the fraction of individuals harboring WBC *BRCA1* methylation to drop with age.[1] In the present study, although we observed a numerical drop in methylated individuals in the highest age groups, we did not detect a significant reduction in methylation as a function of age (eTable 4). Notably, the age span in this study was limited.

eTable 4. Fractions of methylated controls by age at screening

Age group	Methylated	Unmethylated	% methylated
<57	46	743	5.83
≥57 and <62	48	766	5.90
≥62 and <68	53	952	5.27
≥68	47	890	5.02
**total**	**194**	**3351**	**5.47**

Risk estimated for VEF subranges

As a part of sensitivity analysis, we calculated methylation HR using different cutoffs for methylation positivity. Using the subset of samples previously classified as methylation-positive, we calculated VEF quartiles and applied each of them as a new methylation positivity cutoff for the entire dataset. Although, the estimates became somewhat fragile when applying the highest cutoff for HGSOC due to low number of observations (n=9 methylated cases), the five other estimates revealed robust results, similar to the results yielded when applying the main cutoff (eFigure 5).

eFigure 5. Risk of incident TNBC or HGSOC applying increasing methylation cutoff values

A (TNBC)

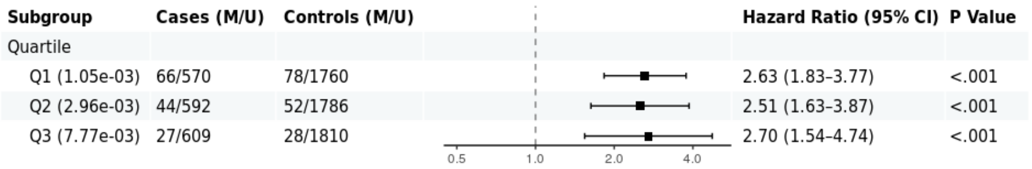

B (HGSOC)

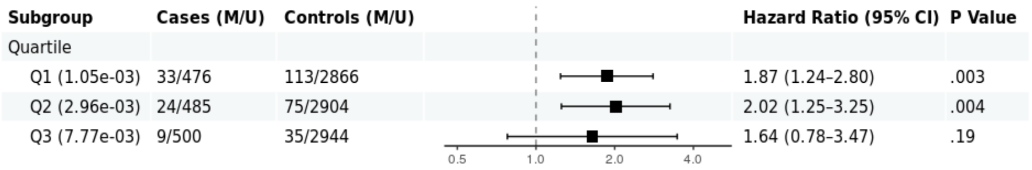

Risk of incident TNBC (A) or HGSOC (B) applying increasing methylation cutoff values. First (Q1), second (Q2) or third (Q3) VEF quartiles were used as a methylation positivity cutoff. The corresponding values are given in parentheses. For this analysis, "quartiles" were defined as quartiles of those samples classified as methylation positive by the main cutoff (see eFigure 4).

© 2022 Lønning PE et al. *JAMA Oncology.*

Risk estimated for optimized methylation positivity cutoffs

In addition to the cutoff applied in all main analyses and in the sensitivity analyses presented in the eResults (eFigure 5), we also sought to identify an optimal cutoff *post-hoc*, based on the methylation distribution in cases and controls. Several optimization strategies were applied, with "MaxDOR" resulting in the highest HRs (3.14 for TNBC and 4.50 for HGSOC), although this approach also resulted in the widest confidence intervals (eFigure 6). Two alternative strategies, "Yoden" and "MinPvalue" resulted in HRs 2.59 and 2.59 for TNBC, and 1.89 and 1.93 for HGSOC, respectively. Thus, these optimizations did not yield very different estimates than our main analyses (HR=2.35 for TNBC and 1.93 for HGSOC; Figures 2 and 3 in the main text).

eFigure 6. Risk of incident TNBC or HGSOC calculated using optimized cutoffs

A (TNBC)

Subgroup	Cases (M/U)	Controls (M/U)	Hazard Ratio (95% CI)	P Value
Optimization method				
Youden (5.77e-04)	79/557	94/1744	2.59 (1.87–3.59)	<.001
MaxDOR (3.37e-03)	42/594	41/1797	3.14 (1.96–5.02)	<.001
MinPvalue (5.77e-04)	79/557	94/1744	2.59 (1.87–3.59)	<.001

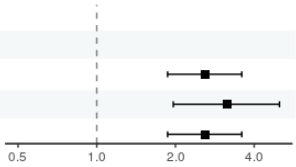

B (HGSOC)

Subgroup	Cases (M/U)	Controls (M/U)	Hazard Ratio (95% CI)	P Value
Optimization method				
Youden (2.90e-04)	50/459	164/2815	1.89 (1.35–2.66)	<.001
MaxDOR (6.15e-02)	2/507	4/2975	4.50 (0.73–27.85)	.11
MinPvalue (3.46e-04)	49/460	159/2820	1.93 (1.37–2.72)	<.001

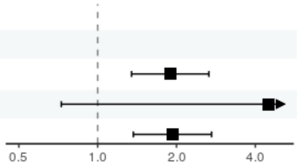

Risk of incident TNBC (A) or HGSOC (B) according to methylation positivity cutoffs for CpG14–34 VEF values, defined by three different optimization strategies: Youden—Youden's Index,[12,13] MaxDOR—maximum Diagnostic Odds Ratio, MinPvalue—minimum p-value associated with the statistical Chi-squared test. Population prevalence was set to 0.015 and 0.006 for TNBC and HGSOC, respectively. Optimal cutoffs obtained are given in parentheses.

Assessing TNBC and HGSOC risk based on methylation of different *BRCA1* promoter regions

Previous studies have applied different technologies and included slightly different sets of CpGs in order to assess *BRCA1* methylation. In our previous study in a hospital-based cohort of ovarian cancer patients,[1] we applied methylation-specific quantitative PCR covering CpG17–18 and 21–29 as well as a pyrosequencing assay covering CpG20–30 (eFigure 1 and eTable 1). To evaluate the potential impact of promoter area selection on our findings we repeated our main analyses on the present data set, restricting it to methylation across the same two sets of CpGs as used in our previous report. Doing so, the obtained results show that the main conclusions are only weakly affected by choice of CpGs (eFigure 7). This indicates the results to be robust, regardless of the choice of CpGs in the core region of the *BRCA1* promoter (i.e., CpG14–34).

eFigure 7. Risk of incident TNBC or HGSOC calculated for different sets of CpGs

A (TNBC)

Subgroup	Cases (M/U)	Controls (M/U)	Hazard Ratio (95% CI)	P Value
Genomic region average				
CpG 17–18, 21–29	79/557	111/1727	2.17 (1.58–2.97)	<.001
CpG 20–30	79/557	108/1730	2.24 (1.63–3.08)	<.001

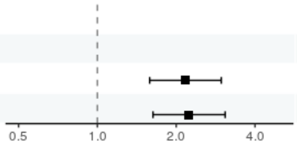

B (HGSOC)

Subgroup	Cases (M/U)	Controls (M/U)	Hazard Ratio (95% CI)	P Value
Genomic region average				
CpG 17–18, 21–29	49/460	169/2810	1.79 (1.27–2.52)	<.001
CpG 20–30	49/460	163/2816	1.88 (1.33–2.65)	<.001

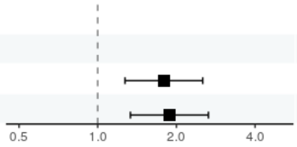

Risk of incident TNBC (A) or HGSOC (B) according to different sets of *BRCA1* promoter CpGs included in analysis. CpG17–18, 21–29 and CpG20–30 mimic quantitative PCR and pyrosequencing assays, respectively, used to assess *BRCA1* promoter methylation in previous studies. The data was assessed by VEF; positivity cutoff values for every region were determined automatically based on probability density of corresponding VEF values, i.e., identical procedure as described above for the region CpG14–34.

© 2022 Lønning PE et al. *JAMA Oncology*.

In addition to the two amplicons used for main analyses, covering the core region of the *BRCA1* promoter (CpG14–34), the present study also included information from two additional amplicons, extending into the gene bodies of *NBR2*[14] and *BRCA1*, and covering CpG00–13 and 33–49, respectively (eFigure 1). In order to assess potential impact of methylation of these regions on the risk of TNBC or HGSOC, we performed subanalyses per amplicon. The results (eFigure 8) revealed that the methylation levels in the amplicon covering CpG33–49 within *BRCA1* gene body had a very similar impact as the two core amplicons, while *NBR2* CpG00–13 did not seem to have the same influence. Notably, in the latter region, most likely not involved in *BRCA1* transcription,[14] a much higher fraction of individuals (~65%) were classified as methylation positive, indicating that methylation in this region is a biologically different feature than methylation in the core of the promoter.

eFigure 8. Risk of incident TNBC or HGSOC calculated for different PCR amplicons

A (TNBC)

Subgroup	Cases (M/U)	Controls (M/U)	Hazard Ratio (95% CI)	P Value
PCR amplicon				
CpG 00–13	417/218	1175/663	1.06 (0.87–1.29)	.54
CpG 14–31	74/562	104/1734	2.19 (1.57–3.04)	<.001
CpG 17–34	75/561	96/1742	2.35 (1.70–3.26)	<.001
CpG 33–49	61/528	78/1616	2.37 (1.64–3.41)	<.001

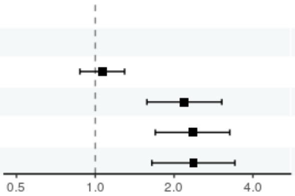

B (HGSOC)

Subgroup	Cases (M/U)	Controls (M/U)	Hazard Ratio (95% CI)	P Value
PCR amplicon				
CpG 00–13	337/172	1905/1073	1.09 (0.89–1.33)	.41
CpG 14–31	42/467	151/2827	1.74 (1.21–2.52)	.003
CpG 17–34	45/464	148/2831	1.85 (1.30–2.64)	<.001
CpG 33–49	38/426	118/2622	2.11 (1.42–3.14)	<.001

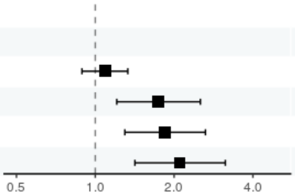

Risk of incident TNBC (A) or HGSOC (B) according to methylation called in four PCR amplicons (assessed by VEF) covering different but partly overlapping regions of the *BRCA1* promoter. Positivity cutoff values for every region were determined automatically based on probability density of corresponding VEF values, i.e., identical procedure as described above for the region CpG14–34.

<u>Other methods for risk estimation</u>

We additionally assessed TNBC and HGSOC risks (odds ratio, OR) using two other statistical methods: crude calculation using 2x2 contingency tables (eFigure 9), and multivariate logistic regression including all covariates (as in the main analyses) but not accounting for matched case-control groups (eFigure 10). Marginal changes in estimates and confidence intervals as compared to the main analyses (Cox proportional hazards regression with covariates in matched case-control groups, Figure 2 and 3 in the main text) were observed. This indicates that choice of statistical method does not influence the results, confirming the robustness of the main biological conclusions.

eFigure 9. Risk (OR) of incident TNBC or HGSOC calculated using 2x2 contingency tables

A (TNBC)

Subgroup	Cases (M/U)	Controls (M/U)	Odds Ratio (95% CI)	P Value
Overall	79/557	104/1734	2.36 (1.71–3.25)	<.001
Age				
≤ median	46/292	57/922	2.55 (1.65–3.91)	<.001
> median	33/265	47/812	2.15 (1.30–3.51)	.002
Race				
White	67/457	89/1429	2.35 (1.66–3.33)	<.001
Other	12/100	15/305	2.43 (1.00–5.78)	.038
Methylated epiallele frequency				
≤ median	35/557	52/1734	2.09 (1.31–3.32)	.001
> median	44/557	52/1734	2.63 (1.70–4.06)	<.001
Years from screening to diagnosis				
≤ 5	16/124	104/1734	2.15 (1.15–3.80)	.01
> 5	63/433	102/1715	2.45 (1.73–3.44)	<.001

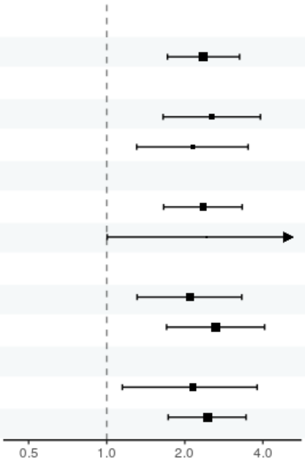

© 2022 Lønning PE et al. *JAMA Oncology*.

B (HGSOC)

Subgroup	Cases (M/U)	Controls (M/U)	Odds Ratio (95% CI)	P Value
Overall	48/461	156/2823	1.88 (1.31–2.66)	<.001
Age				
≤ median	29/245	88/1409	1.89 (1.17–2.98)	.008
> median	19/216	68/1414	1.83 (1.02–3.15)	.036
Race				
White	46/431	144/2631	1.95 (1.35–2.78)	<.001
Other	2/30	12/192	1.07 (0.11–5.16)	1
Methylated epiallele frequency				
≤ median	24/461	81/2823	1.81 (1.09–2.93)	.016
> median	24/461	75/2823	1.96 (1.17–3.18)	.008
Years from screening to diagnosis				
≤ 5	13/113	156/2823	2.08 (1.05–3.81)	.024
> 5	35/348	153/2778	1.83 (1.21–2.70)	.003

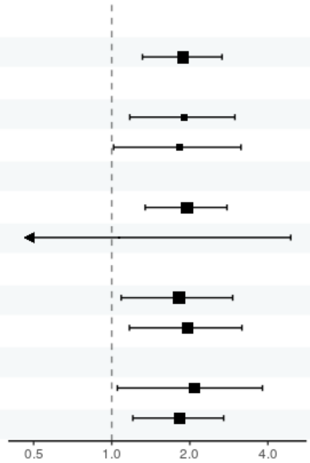

Risk of incident TNBC (A) or HGSOC (B) applying the same data as in Figure 2 and 3 of the main text, but with odds ratios (ORs) and CIs calculated using 2x2 contingency tables without correction for covariates.

eFigure 10. Risk (OR) of incident TNBC or HGSOC calculated using multivariate logistic regression

A (TNBC)

Subgroup	Cases (M/U)	Controls (M/U)	Odds Ratio (95% CI)	P Value
Overall	79/557	104/1734	2.44 (1.79–3.33)	<.001
Age				
≤ median	46/292	57/922	2.62 (1.73–3.96)	<.001
> median	33/265	47/812	2.25 (1.39–3.62)	<.001
Race				
White	67/457	89/1429	2.43 (1.73–3.40)	<.001
Other	12/100	15/305	2.57 (1.13–5.75)	.022
Methylated epiallele frequency				
≤ median	35/557	52/1734	2.16 (1.38–3.35)	<.001
> median	44/557	52/1734	2.73 (1.79–4.14)	<.001
Years from screening to diagnosis				
≤ 5	16/124	104/1734	2.34 (1.28–4.04)	.003
> 5	63/433	102/1715	2.53 (1.81–3.54)	<.001

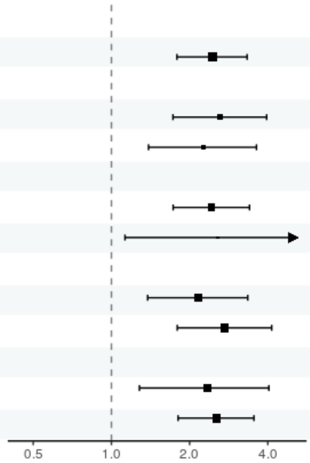

B (HGSOC)

Subgroup	Cases (M/U)	Controls (M/U)	Odds Ratio (95% CI)	P Value
Overall	48/461	156/2823	1.87 (1.32–2.61)	<.001
Age				
≤ median	29/245	88/1409	1.90 (1.20–2.93)	.005
> median	19/216	68/1414	1.83 (1.04–3.05)	.027
Race				
White	46/431	144/2631	1.94 (1.36–2.73)	<.001
Other	2/30	12/192	1.14 (0.16–4.89)	.87
Methylated epiallele frequency				
≤ median	24/461	81/2823	1.79 (1.10–2.82)	.014
> median	24/461	75/2823	1.96 (1.20–3.09)	.005
Years from screening to diagnosis				
≤ 5	13/113	156/2823	2.15 (1.13–3.78)	.013
> 5	35/348	153/2778	1.81 (1.21–2.63)	.003

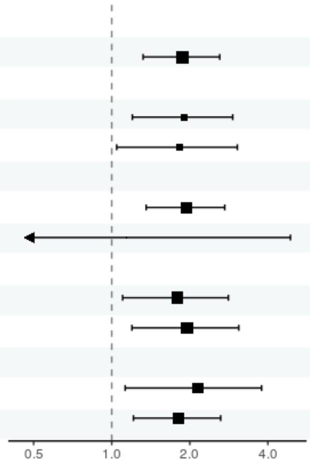

Risk of incident TNBC (A) or HGSOC (B) applying the same data as in Figure 2 and 3 of the main text but with ORs and CIs calculated using multivariate logistic regression accounting for all covariates without stratification according to case-control matched groups.

© 2022 Lønning PE et al. *JAMA Oncology*.

Methylation beta values

For additional sensitivity analysis we calculated TNBC and HGSOC risk using methylation beta values as a metric for DNA methylation.

Positivity cutoffs for the methylation beta values averaged for the region CpG14–34 (equals 7.88e-03 and 7.91e-03 for TNBC and HGSOC, respectively) were defined as the most optimal cutoff values based on Youden optimization method (as described in the eMethods).[12,13] Comparing beta values to VEF, we found a strong correlation between these two measures in the high range of values (upper right area of the eFigure 11). However, a fraction of samples defined as methylation-positive by VEF were classified as methylation-negative by beta values (upper left area of the eFigure 11). As such, in addition to the biological relevance of fully methylated reads/alleles, also from a pure technical perspective, we found application of VEF to be superior to beta values for methylation positivity calling.

The superiority of VEF as a measure for *BRCA1* methylation was established prior to unblinding case-control status and led to an amendment of the study protocol (see the Study protocol).

eFigure 11. Scatter plot of methylation beta and VEF values

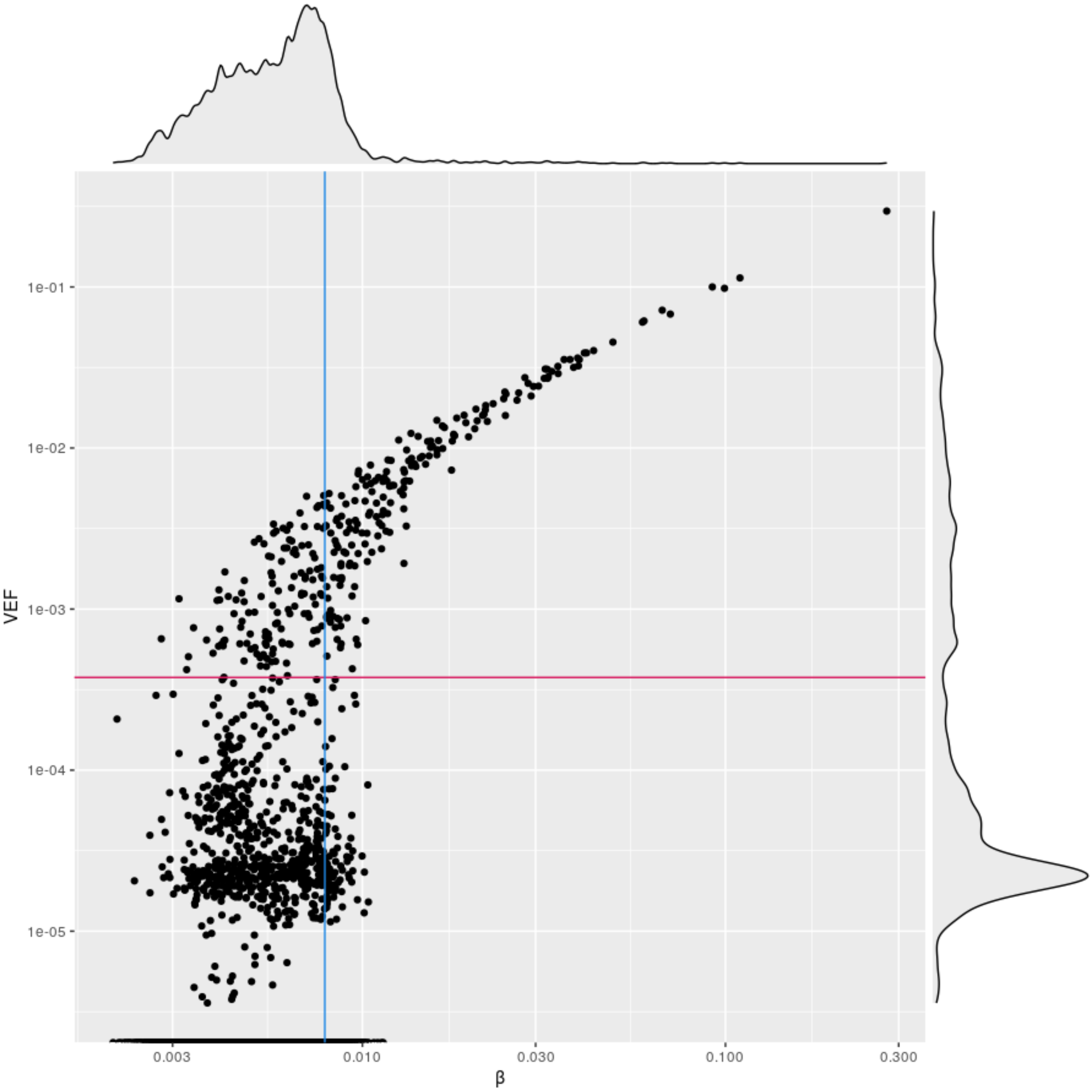

Scatter plot and corresponding probability density plots showing the relationship between methylation beta (x-axis) and VEF (y-axis) values for all samples (cases and controls) analyzed in the present study. The VEF cutoff value applied for all main analyses is represented by the horizontal pink line. Nearly identical cutoffs for the methylation beta values for TNBC and HGSOC are represented by the single blue line.

Despite these limitations of using beta values, we found all main and subanalyses for TNBC to be very similar to the ones yielded by application of VEF (eFigure 12). The main estimate (HR 2.45 [95% CI 1.75–3.43]) as well as the majority of subanalyses were marginally affected. For HGSOC, the results were also similar: the main estimate

© 2022 Lønning PE et al. *JAMA Oncology*.

was moderately weakened (from HR 1.93 [95% CI 1.36–2.73] to 1.61 [1.23–2.12]), and one of subanalyses lost statistical significance. Taken together, the results from this sensitivity analysis support the main conclusion.

eFigure 12. Risk of incident TNBC or HGSOC calculated using beta instead of VEF values

A (TNBC)

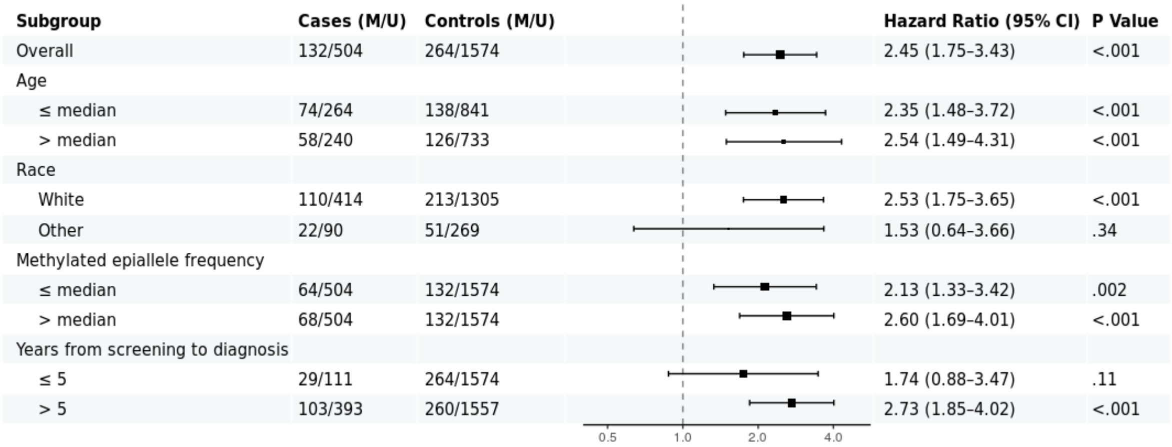

Subgroup	Cases (M/U)	Controls (M/U)	Hazard Ratio (95% CI)	P Value
Overall	132/504	264/1574	2.45 (1.75-3.43)	<.001
Age				
≤ median	74/264	138/841	2.35 (1.48-3.72)	<.001
> median	58/240	126/733	2.54 (1.49-4.31)	<.001
Race				
White	110/414	213/1305	2.53 (1.75-3.65)	<.001
Other	22/90	51/269	1.53 (0.64-3.66)	.34
Methylated epiallele frequency				
≤ median	64/504	132/1574	2.13 (1.33-3.42)	.002
> median	68/504	132/1574	2.60 (1.69-4.01)	<.001
Years from screening to diagnosis				
≤ 5	29/111	264/1574	1.74 (0.88-3.47)	.11
> 5	103/393	260/1557	2.73 (1.85-4.02)	<.001

B (HGSOC)

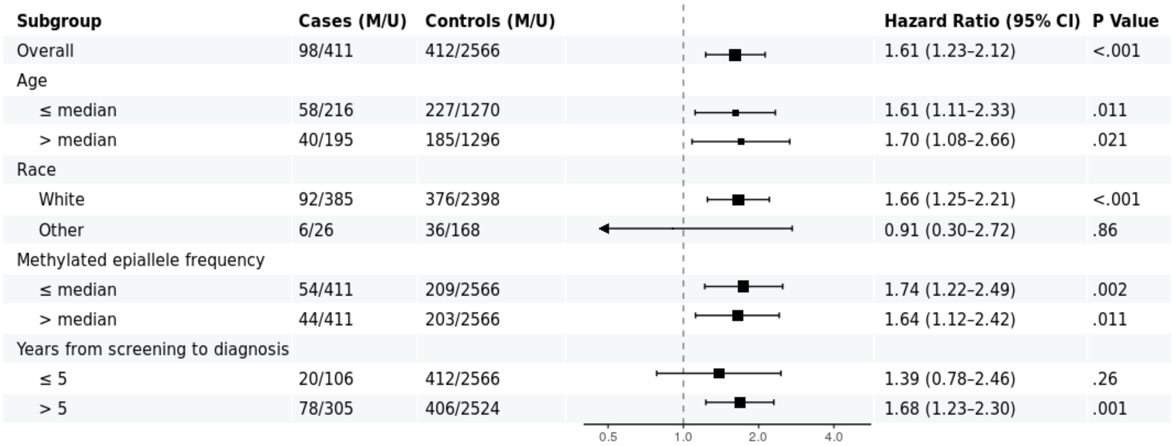

Subgroup	Cases (M/U)	Controls (M/U)	Hazard Ratio (95% CI)	P Value
Overall	98/411	412/2566	1.61 (1.23-2.12)	<.001
Age				
≤ median	58/216	227/1270	1.61 (1.11-2.33)	.011
> median	40/195	185/1296	1.70 (1.08-2.66)	.021
Race				
White	92/385	376/2398	1.66 (1.25-2.21)	<.001
Other	6/26	36/168	0.91 (0.30-2.72)	.86
Methylated epiallele frequency				
≤ median	54/411	209/2566	1.74 (1.22-2.49)	.002
> median	44/411	203/2566	1.64 (1.12-2.42)	.011
Years from screening to diagnosis				
≤ 5	20/106	412/2566	1.39 (0.78-2.46)	.26
> 5	78/305	406/2524	1.68 (1.23-2.30)	.001

Risk of incident TNBC (A) or HGSOC (B) associated with *BRCA1* methylation as scored by beta values.

<u>Risk connected to previous hormone use</u>

In an exploratory *post-hoc* analysis we estimated risk of incident TNBC and HGSOC for carriers of methylated *BRCA1* epialleles within subgroups defined by the hormone use prior to enrolment. Noteworthy, this was a non-preplanned analysis, and the sample set was not powered specifically for addressing this issue.

An elevated risk for incident HGSOC linked to *BRCA1* methylation was observed in individuals who had used estrogens alone (eFigure 13). The follow-up analysis showed a nominally significant interaction for estrogen-only use with methylation-positive status (HGSOC only; P=0.030; HR ratio of 2.72; 95% CI, 1.10 to 6.70), while other possible interactions remained nonsignificant. Importantly, the overall risk estimates for TNBC and HGSOC, with respect to *BRCA1* methylation, were in the same range even when accounting for the interaction between hormone use and *BRCA1* methylation (HR 2.78; 95% CI, 1.73 to 4.46; and HR 2.01; 95% CI, 1.13 to 3.57; for TNBC and HGSOC, respectively; eFigure 14). Of note, the fraction of methylation carriers in the hormone therapy groups were roughly the same (eTable 5).

© 2022 Lønning PE et al. *JAMA Oncology*.

eFigure 13. Risk of incident TNBC or HGSOC in subgroups based on hormone therapy exposure

A (TNBC)

Subgroup	Cases (M/U)	Controls (M/U)	Hazard Ratio (95% CI)	P Value
Hormone use				
no hormone therapy	37/229	40/739	3.54 (2.04–6.17)	<.001
estrogen	20/175	24/510	3.47 (1.61–7.47)	.001
estrogen and progestin	22/153	40/485	1.93 (0.98–3.81)	.057

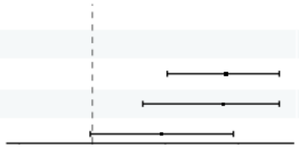

B (HGSOC)

Subgroup	Cases (M/U)	Controls (M/U)	Hazard Ratio (95% CI)	P Value
Hormone use				
no hormone therapy	18/176	64/1207	1.39 (0.71–2.71)	.34
estrogen	18/109	22/681	5.80 (2.73–12.33)	<.001
estrogen and progestin	12/176	70/935	0.95 (0.49–1.83)	.88

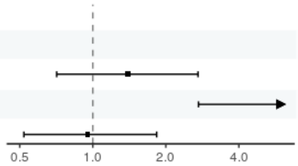

Risk of incident TNBC (A) or HGSOC (B) associated with *BRCA1* promoter methylation (assessed by CpG14–34 VEF) in hormone therapy groups.

eFigure 14. Risk of incident TNBC or HGSOC accounting for potential interaction between methylation and hormone therapy

Disease	Cases (M/U)	Controls (M/U)	Hazard Ratio (95% CI)	P Value
TNBC	79/557	104/1734	2.78 (1.73–4.46)	<.001
HGSOC	48/461	156/2823	2.01 (1.13–3.57)	.017

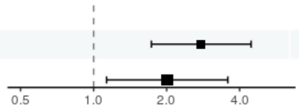

Risk of incident TNBC or HGSOC associated with *BRCA1* promoter methylation (assessed by CpG14–34 VEF) accounting for potential interaction between methylation and hormone therapy.

eTable 5. Fractions of methylated *BRCA1* epiallele carriers by hormone therapy group

Group	Methylated	Unmethylated	% methylated
No therapy	131	1812	6.74
Oestrogen alone	77	1208	5.99
Oestrogen and progestin	112	1345	7.69

<u>Risk in other subgroups</u>

As an additional sensitivity analysis, we performed risk estimation in subgroups of samples excluding those diagnosed with both TNBC and HGSOC (n=5), or subjects to bilateral oophorectomy (TNBC cases and controls only, n=438). Overall risk estimates were in the same range, which confirms our main conclusions (eFigure 15).

eFigure 15. Risk of incident TNBC or HGSOC calculated for subsets of samples

A (TNBC)

Subgroup	Cases (M/U)	Controls (M/U)	Hazard Ratio (95% CI)	P Value
Samples removed				
samples with TNBC+HGSOC removed	78/553	104/1734	2.33 (1.69–3.21)	<.001
samples with oophorectomy removed	64/444	79/1412	2.47 (1.72–3.53)	<.001

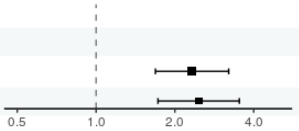

B (HGSOC)

Subgroup	Cases (M/U)	Controls (M/U)	Hazard Ratio (95% CI)	P Value
Samples removed				
samples with TNBC+HGSOC removed	47/457	156/2823	1.90 (1.34–2.70)	<.001

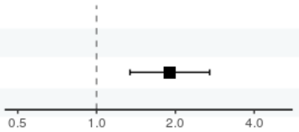

Risk of incident TNBC (A) or HGSOC (B) associated with *BRCA1* promoter methylation in samples excluding double-diagnosed cases or individuals that undergone oophorectomy (TNBC only).

© 2022 Lønning PE et al. *JAMA Oncology*.

Allele specificity of methylation

The sequenced region contains a single-nucleotide polymorphism (SNP; rs799905) with relatively high variant allele frequency. rs799905 is located in the flanking amplicon that covers CpGs 33–49 and extends into the *BRCA1* gene body (eFigure 1). We counted the methylated and unmethylated reads carrying reference or alternative SNP alleles in the subset of samples defined as methylation-positive in the region CpG33–49 (same subset as defined as positive in eFigure 8), and thereby assessed the specificity of DNA methylation in the region CpG33–49 with regard to the allelic status of the SNP.

Assessing these data across individuals, we did not observe any allelic preference for methylation (eTable 6). This was in line with the previous finding that *BRCA1* promoter methylation was equally distributed in both ancient *BRCA1* haplotypes.[1] However, assessing the same data intraindividually, methylated reads from heterozygous samples preferentially contained only one allele (eFigure 16). In >90% of individuals, >95% of the methylated reads were linked to one of the rs799905 alleles (either the reference *or* the alternative rs799905-allele, per individual). This striking observation indicates that the *BRCA1* methylation was likely acquired during early development followed by clonal expansion.

eTable 6. Sample counts in relation to rs799905 SNP genotype

Genotype<br>Category	REF/REF	REF/ALT	ALT/ALT
Methylated	101 (5.0%)	108 (5.6%)	37 (5.1%)
Total	2022	1939	731

eFigure 16. Allele specificity of methylation

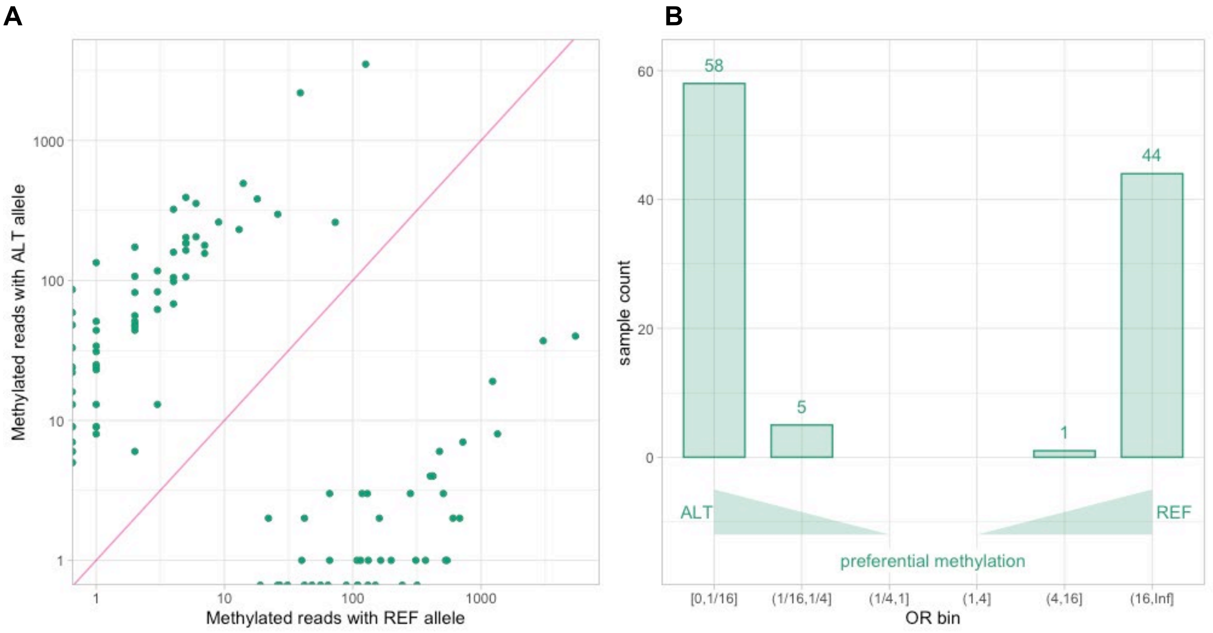

(A) Counts of methylated reads carrying reference (x-axis) or alternative (y-axis) allele of the SNP rs799905 for all methylated samples heterozygous for rs799905 (n=108). (B) Distribution (histogram) of allele-specific methylation assessed as odds ratio (OR). OR for association between SNP allele and methylation status was calculated as $REF^{meth} \times ALT^{unmeth} / REF^{unmeth} \times ALT^{meth}$ for all heterozygous methylated samples, where X^{meth} and X^{unmeth} are the number of methylated or unmethylated reads, carrying reference (REF) or alternative (ALT) SNP allele, respectively. OR>1 if reference SNP allele occurs more often within methylated strands. Numbers above each bar indicate sample counts within the corresponding OR bin.

BRCA1 promoter methylation in *BRCA1* germline mutation carriers

The WHI ancillary study AS508 characterized prevalence and penetrance of breast cancer-associated mutations using a subset of 2,500 incident breast cancer cases and 2,500 controls.[15] There, next-generation sequencing and large rearrangement analysis was performed using a panel of 28 genes; among these, *BRCA1/2*, *ATM*, *BARD1*, *CDH1*, *CHEK2*, *NBN*, *PALB2*, *STK11*, and *TP53* were considered breast cancer associated (full list of genes is given in eTable 7).

Overlapping these data with our set of samples, we identified 234 shared cases and controls, of which 88 were germline mutation carriers in at least one gene (eTable 7). We did not observe significantly increased

© 2022 Lønning PE et al. *JAMA Oncology*.

methylation in either mutation carriers or noncarriers, moreover, none of the *BRCA1* (n = 6) or *BRCA2* (n = 2) germline mutation carriers were classified as *BRCA1* promoter methylated. This argues against co-occurrence of germline mutations and allelic methylation within *BRCA1* and supports our previous observation that these two events are mutually exclusive in patients diagnosed with HGSOC.[1] Moreover, we observed no significant positive association between *BRCA1* methylation and germline mutations in any of the other 28 genes included in the panel.

eTable 7. Distribution of germline mutations in shared case and control samples

**Sample methylation** / **Gene**	**TNBC cases**		**HGSOC cases**		**Controls**	
	**Unmeth**	**Meth**	**Unmeth**	**Meth**	**Unmeth**	**Meth**
APC	7 (1)*	0	0	0	0	0
ATM	5 (1)	1 (0)	0	0	1 (0)	0
BARD1	5 (1)	1 (0)	0	0	0	0
**BRCA1**	**5 (2)**	**0**	**1 (1)**	**0**	**0**	**0**
**BRCA2**	**1 (1)**	**0**	**1 (1)**	**0**	**0**	**0**
BRIP1	1 (0)	0	0	0	1 (0)	0
CDH1	3 (0)	1 (0)	0	0	0	0
CDK4	1 (0)	0	0	0	1 (0)	1 (0)
CHEK2	1 (0)	0	0	0	1 (0)	0
MLH1	1 (0)	0	0	0	0	0
MSH2	2 (0)	0	0	0	1 (0)	0
MSH6	1 (0)	1 (0)	0	0	1 (0)	0
MYH	6 (4)	0	0	0	4 (2)	0
NBN	6 (0)	0	0	0	3 (0)	1 (0)
P14ARF	1 (0)	0	0	0	0	0
P16	6 (0)	1 (1)	0	0	0	0
PALB2	1 (1)	0	0	0	2 (0)	0
PMS2	1 (0)	1 (0)	0	0	0	0
POLD1	4 (0)	0	0	0	1 (0)	0
POLE	5 (0)	1 (0)	0	0	1 (0)	1 (0)
PTEN	1 (0)	0	0	0	0	0
RAD51C	5 (2)	0	0	0	1 (0)	0
RAD51D	2 (0)	0	0	0	0	0
SMAD4	0	0	0	0	1 (0)	0
STK11	1 (0)	0	0	0	0	0
**Total (carriers)**	**72 (13)**	**7 (1)**	**2 (2)**	**0**	**19 (2)**	**3 (0)**
Failed	5	1	0	0	1	0
No Mutation Detected	87	14	4	0	42	0
**Total (all)**	**164**	**22**	**6**	**0**	**62**	**3**

* Number of individuals carrying deleterious or suspected deleterious mutations are given in parentheses

© 2022 Lønning PE et al. *JAMA Oncology*.

eReferences

1 Lønning PE, Berge EO, Bjørnslett M, *et al.* White Blood Cell BRCA1 Promoter Methylation Status and Ovarian Cancer Risk. *Ann Intern Med* 2018; **168**: 326–34.

2 Nikolaienko O, Lønning PE, Knappskog S. epialleleR: an R/BioC package for sensitive allele-specific methylation analysis in NGS data. 2022; : 2022.06.30.498213.

3 López-Ratón M, Rodríguez-Álvarez MX, Cadarso-Suárez C, Gude-Sampedro F. OptimalCutpoints: An R Package for Selecting Optimal Cutpoints in Diagnostic Tests. *Journal of Statistical Software* 2014; **61**: 1–36.

4 Huh I, Wu X, Park T, Yi SV. Detecting differential DNA methylation from sequencing of bisulfite converted DNA of diverse species. *Briefings in Bioinformatics* 2019; **20**: 33–46.

5 Youk J, An Y, Park S, Lee J-K, Ju YS. The genome-wide landscape of C:G > T:A polymorphism at the CpG contexts in the human population. *BMC Genomics* 2020; **21**: 270.

6 Warnecke PM, Stirzaker C, Melki JR, Millar DS, Paul CL, Clark SJ. Detection and measurement of PCR bias in quantitative methylation analysis of bisulphite-treated DNA. *Nucleic Acids Research* 1997; **25**: 4422–6.

7 Moskalev EA, Zavgorodnij MG, Majorova SP, *et al.* Correction of PCR-bias in quantitative DNA methylation studies by means of cubic polynomial regression. *Nucleic Acids Research* 2011; **39**: e77–e77.

8 Prajzendanc K, Domagała P, Hybiak J, *et al.* BRCA1 promoter methylation in peripheral blood is associated with the risk of triple-negative breast cancer. *International Journal of Cancer* 2020; **146**: 1293–8.

9 Kint S, Spiegelaere WD, Kesel JD, Vandekerckhove L, Criekinge WV. Evaluation of bisulfite kits for DNA methylation profiling in terms of DNA fragmentation and DNA recovery using digital PCR. *PLOS ONE* 2018; **13**: e0199091.

10 Costello M, Fleharty M, Abreu J, *et al.* Characterization and remediation of sample index swaps by non-redundant dual indexing on massively parallel sequencing platforms. *BMC Genomics* 2018; **19**: 332.

11 Sinha R, Stanley G, Gulati GS, *et al.* Index switching causes “spreading-of-signal” among multiplexed samples in Illumina HiSeq 4000 DNA sequencing. 2017.

12 Youden WJ. Index for rating diagnostic tests. *Cancer* 1950; **3**: 32–5.

13 Greiner M, Pfeiffer D, Smith RD. Principles and practical application of the receiver-operating characteristic analysis for diagnostic tests. *Prev Vet Med* 2000; **45**: 23–41.

14 Wang T, Li Z, Yan L, Yan F, Shen H, Tian X. Long Non-Coding RNA Neighbor of BRCA1 Gene 2: A Crucial Regulator in Cancer Biology. *Frontiers in Oncology* 2021; **11**: 5142.

15 Kurian AW, Bernhisel R, Larson K, *et al.* Prevalence of Pathogenic Variants in Cancer Susceptibility Genes Among Women With Postmenopausal Breast Cancer. *JAMA* 2020; **323**: 995–7.

© 2022 Lønning PE et al. *JAMA Oncology*.
